# Supplementary material for: RYBP regulates selective genomic binding of TrxG and PcG components in embryonic stem cell fate control
Source: EMBO J. 2026 Apr 28;45(11):3808–32. doi: 10.1038/s44318-026-00788-y (PMC13226663; doi:10.1038/s44318-026-00788-y)
Supplement: Supplementary file 1 — Appendix [file 44318_2026_788_MOESM1_ESM.pdf]

APPENDIX

Table of Contents:

**Appendix Figure S1**	Page 2
**Appendix Figure S2**	Page 5
**Appendix Figure S3**	Page 8
**Appendix Figure S4**	Page 10
**Appendix Figure S5**	Page 13
**Appendix Figure S6**	Page 15
**Appendix Table S1**	Page 17
**Appendix Table S2**	Page 18

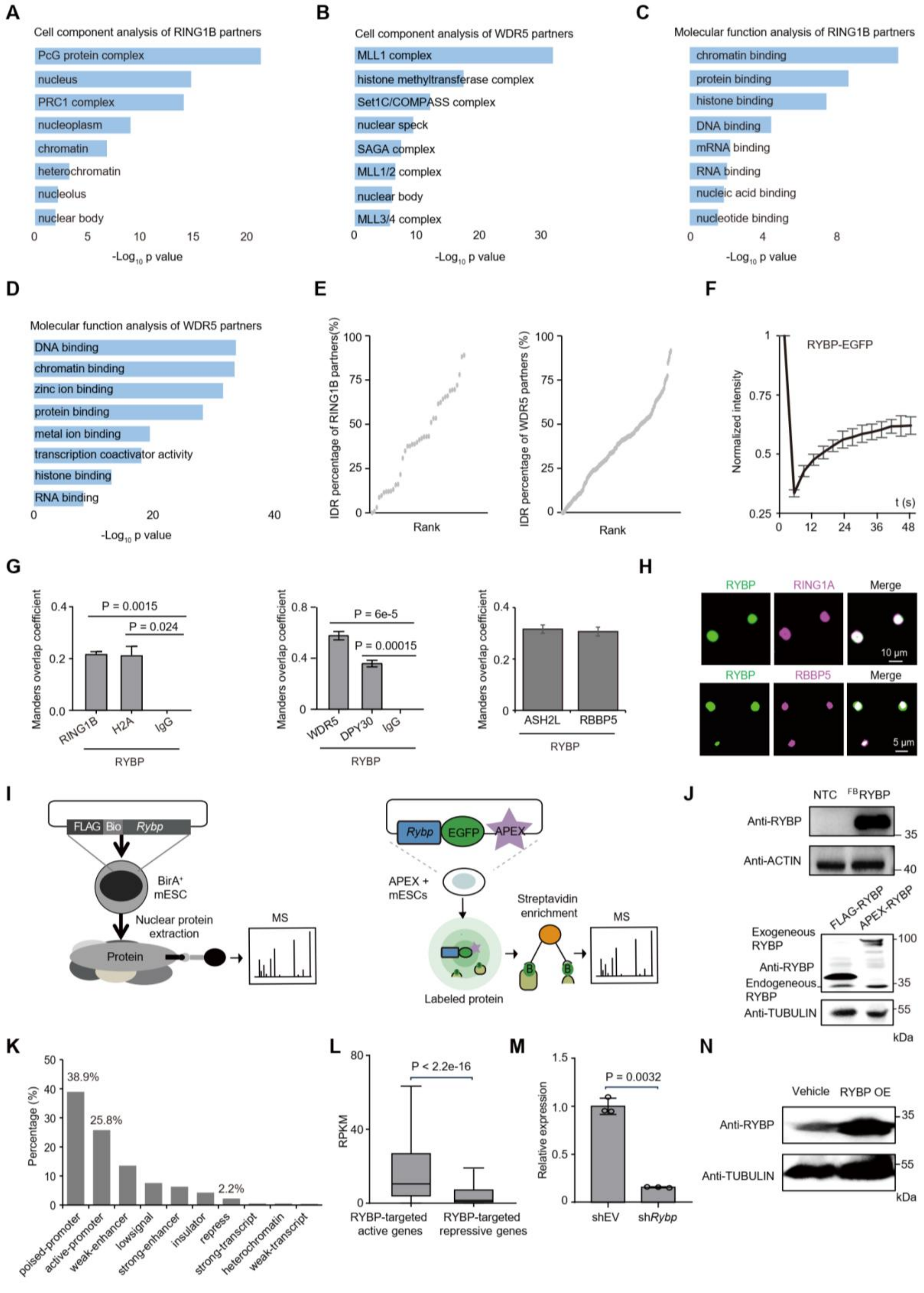
A
Cell component analysis of RING1B partners
PcG protein complex
nucleus
PRC1 complex
nucleoplasm
chromatin
heterochromatin
nucleolus
nuclear body
-Log10 p value
B
Cell component analysis of WDR5 partners
MLL1 complex
histone methyltransferase complex
Set1C/COMPASS complex
nuclear speck
SAGA complex
MLL1/2 complex
nuclear body
MLL3/4 complex
-Log10 p value
C
Molecular function analysis of RING1B partners
chromatin binding
protein binding
histone binding
DNA binding
mRNA binding
RNA binding
nucleic acid binding
nucleotide binding
-Log10 p value
D
Molecular function analysis of WDR5 partners
DNA binding
chromatin binding
zinc ion binding
protein binding
metal ion binding
transcription coactivator activity
histone binding
RNA binding
-Log10 p value
E
IDR percentage of RING1B partners(%)
Rank
IDR percentage of WDR5 partners (%)
Rank
F
RYBP-EGFP
Normalized intensity
t (s)
G
Manders overlap coefficient
P = 0.0015
P = 0.024
RING1B
H2A
IgG
RYBP
P = 6e-5
P = 0.00015
WDR5
DPY30
IgG
RYBP
ASH2L
RBBP5
RYBP
H
RYBP
RING1A
Merge
10 μm
RYBP
RBBP5
Merge
5 μm
I
FLAG
Bio
Rybp
BirA+ mESC
Nuclear protein extraction
Protein
MS
Rybp
EGFP
APEX
APEX + mESCs
Streptavidin enrichment
MS
Labeled protein
J
NTC
FBRYBP
Anti-RYBP
Anti-ACTIN
FLAG-RYBP
APEX-RYBP
Exogeneous RYBP
Anti-RYBP
Endogeneous RYBP
Anti-TUBULIN
kDa
K
Percentage (%)
38.9%
25.8%
2.2%
poised-promoter
active-promoter
weak-enhancer
lowsignal
strong-enhancer
insulator
repress
strong-transcript
heterochromatin
weak-transcript
L
P < 2.2e-16
RPKM
RYBP-targeted active genes
RYBP-targeted repressive genes
M
P = 0.0032
Relative expression
shEV
shRybp
N
Vehicle
RYBP OE
Anti-RYBP
Anti-TUBULIN
kDa

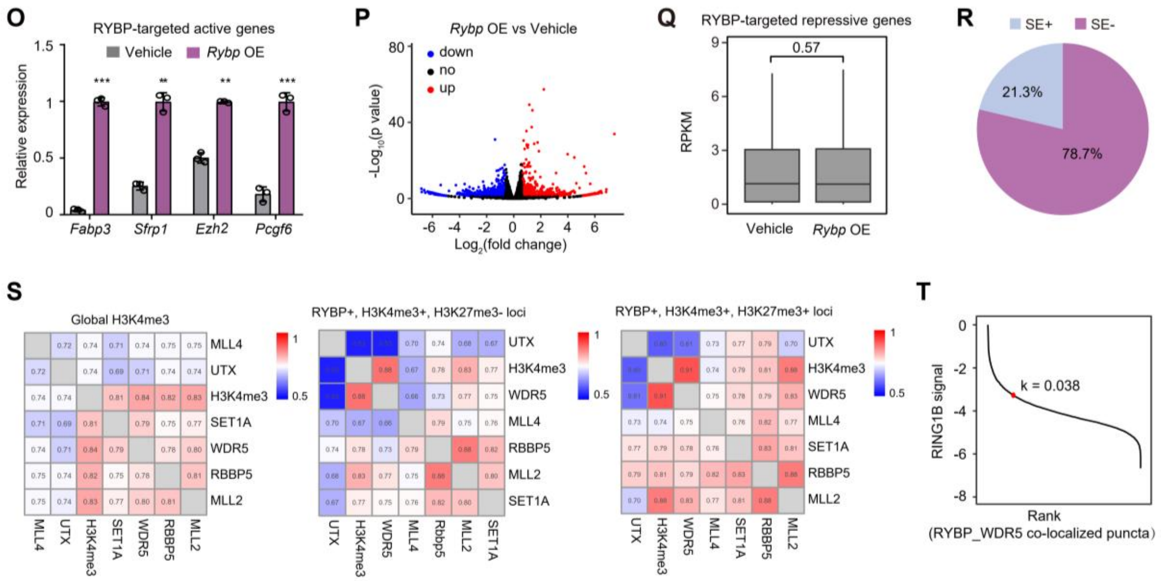

Appendix Figure S1. RYBP is involved in both transcriptionally active and repressive functions.

(**A, B**) Gene ontology analysis showing the cell components of RING1B and WDR5 partners. (**C, D**) Gene ontology analysis showing the molecular function of RING1B and WDR5 partners. (**E**) IDR percentage analysis of RING1B and WDR5 partners. (**F**) Quantification of fluorescence recovery after photobleaching (FRAP) in mESCs expressing exogenous RYBP-EGFP. Data are plotted as means ± SEM, n = 3. (**G**) Quantification showing the co-localization between RYBP and RING1B, RYBP and H2AK119ub1 (H2A), RYBP and WDR5, RYBP and DPY30, RYBP and ASH2L, RYBP and RBBP5, Two-tailed Welch's t-test, cell numbers are (from left to right): n = 80 cells, n = 46 cells, n = 56 cells, n = 104 cells, n = 163 cells, n = 50 cells, n = 145 cells, n = 130 cells, presented as the mean ± SEM. (**H**) RYBP droplets incorporate PcG component (RING1A) and TrxG component (RBBP5) protein *in vitro*. (**I**) IP-MS (Left) and APEX-mediated proximity labeling (Right) schematic diagram for identifying RYBP protein interactome. (**J**) Western blot showing the exogenous expression of biotin-tagged RYBP (^{FB}RYBP) (Top) and FLAG-tagged RYBP-APEX (Bottom). (**K**) Genomic distribution of RYBP binding sites. (**L**) Boxplot showing expression of genes at RYBP-targeted active and RYBP-targeted repressive loci. One-tailed Wilcoxon, n values are (from left to right): 4257 and 3089, data are presented as box plots showing the median (centre line), the

25th and 75th percentiles (box limits), and the minimum and maximum values (defined as the whisker ends, which extend to data points within 1.5 times the interquartile range from the box). (**M**) RT-qPCR showing the efficiency of *Rybp* knockdown in ESCs. Two-tailed Welch's t-test; n = 3 for the two groups, presented as the mean ± SD. (**N**) Western blot showing the overexpression levels of RYBP in ESCs. (**O**) The expression changes of RYBP-targeted active genes after RYBP overexpression. Two-tailed Welch's t-test, all n values are 3; P values are (from left to right): 9.4e-05, 0.0011, 0.0021 and 0.0002, presented as the mean ± SD. (**P**) Volcano plot showing the gene alteration upon RYBP overexpression. (**Q**) Boxplot showing expression change of RYBP-targeted repressive genes upon RYBP overexpression, Two-tailed Wilcoxon, n = 3089, data are presented as box plots showing the median (centre line), the 25th and 75th percentiles (box limits), and the minimum and maximum values (defined as the whisker ends, which extend to data points within 1.5 times the interquartile range from the box). (**R**) RYBP-targeted active sites also occupied by WDR5, the percentage of these peaks localized to SE regions (SE+) or non-SE regions (SE-). (**S**) Correlation analysis of TrxG components with H3K4me3 at different loci. (**T**) Signal distribution of RING1B in RYBP_WDR5 co-localized puncta, red dots indicating the loci with obvious change of intensity. **P < 0.01, ***P < 0.001.

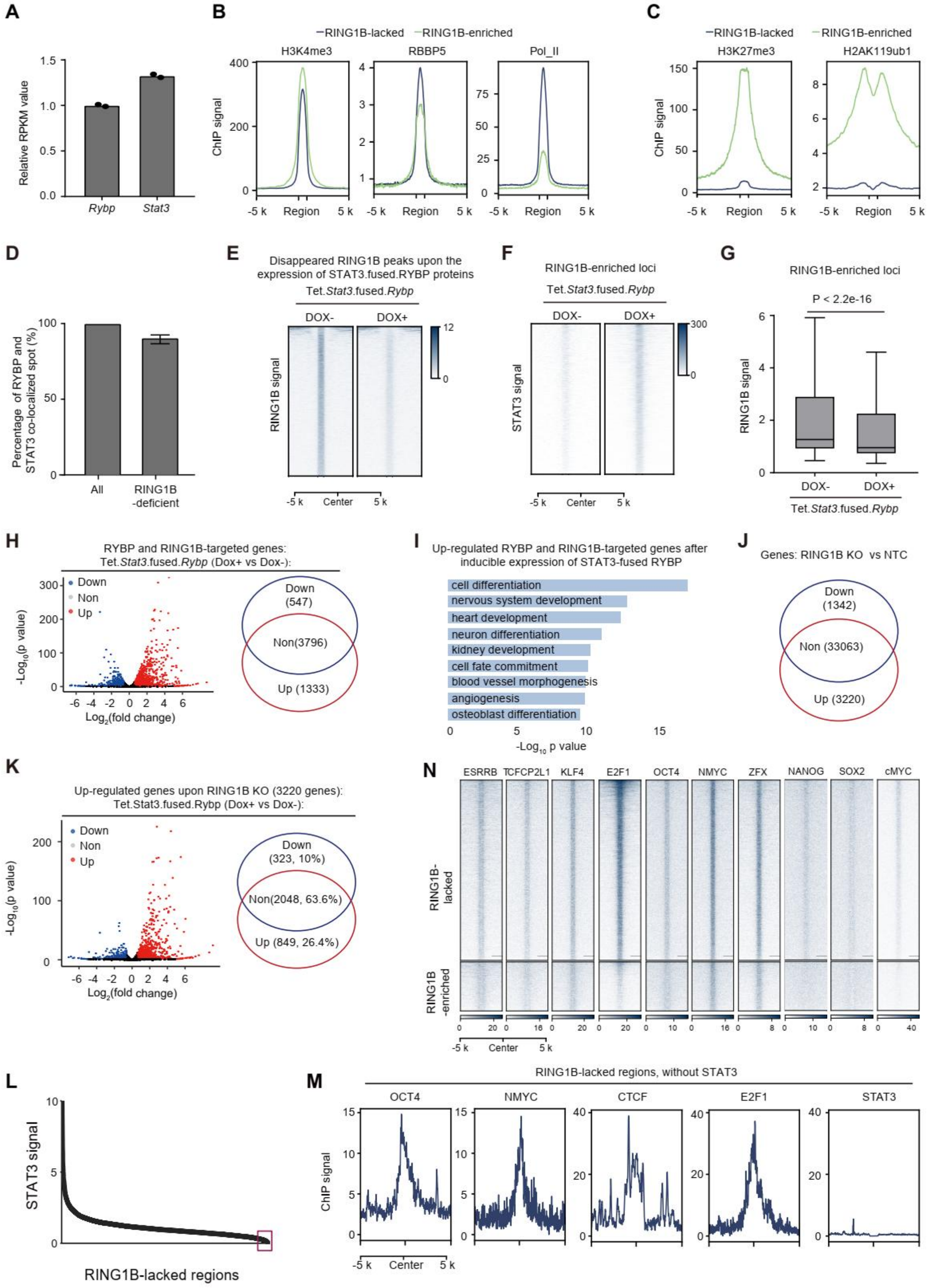
A
Relative RPKM value
1.5
1.0
0.5
0.0
Rybp
Stat3
B
RING1B-lacked
RING1B-enriched
H3K4me3
RBBP5
Pol_II
ChIP signal
-5 k
Region
5 k
C
RING1B-lacked
RING1B-enriched
H3K27me3
H2AK119ub1
ChIP signal
D
Percentage of RYBP and STAT3 co-localized spot (%)
All
RING1B -deficient
E
Disappeared RING1B peaks upon the expression of STAT3.fused.RYBP proteins
Tet.Stat3.fused.Rybp
DOX-
DOX+
RING1B signal
-5 k
Center
5 k
F
RING1B-enriched loci
Tet.Stat3.fused.Rybp
DOX-
DOX+
STAT3 signal
G
RING1B-enriched loci
P < 2.2e-16
RING1B signal
DOX-
DOX+
Tet.Stat3.fused.Rybp
H
RYBP and RING1B-targeted genes:
Tet.Stat3.fused.Rybp (Dox+ vs Dox-):
Down
Non
Up
-Log10(p value)
Log2(fold change)
Down (547)
Non(3796)
Up (1333)
I
Up-regulated RYBP and RING1B-targeted genes after inducible expression of STAT3-fused RYBP
cell differentiation
nervous system development
heart development
neuron differentiation
kidney development
cell fate commitment
blood vessel morphogenesis
angiogenesis
osteoblast differentiation
-Log10 p value
J
Genes: RING1B KO vs NTC
Down (1342)
Non (33063)
Up (3220)
K
Up-regulated genes upon RING1B KO (3220 genes):
Tet.Stat3.fused.Rybp (Dox+ vs Dox-):
Down
Non
Up
-Log10(p value)
Log2(fold change)
Down (323, 10%)
Non(2048, 63.6%)
Up (849, 26.4%)
N
ESRRB
TCFCP2L1
KLF4
E2F1
OCT4
NMYC
ZFX
NANOG
SOX2
cMYC
RING1B-lacked
RING1B -enriched
-5 k
Center
5 k
L
STAT3 signal
RING1B-lacked regions
M
RING1B-lacked regions, without STAT3
OCT4
NMYC
CTCF
E2F1
STAT3
ChIP signal
-5 k
Center
5 k

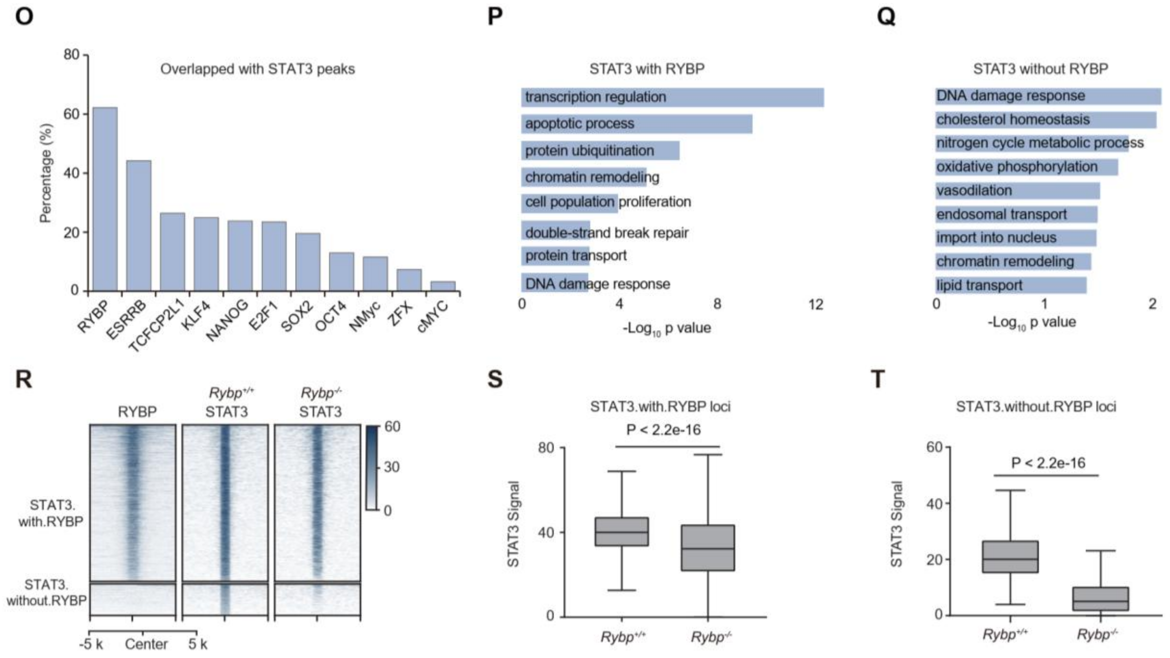

Appendix Figure S2. STAT3 excludes RING1B on chromatin.

(**A**) The histogram showing the relative RPKM value of *Rybp* and *Stat3*, n = 2. (**B, C**) ChIP-seq signal at RING1B-lacked and RING1B-enriched regions. (**D**) The histogram showing the percentage of RYBP and STAT3 co-localized spots without RING1B, n (spot number) is 579, presented as the mean ± SD. (**E**) Heatmap showing the ChIP signal of RING1B at disappeared RING1B peak loci upon inducing expression of STAT3-fused RYBP protein. (**F**) Heatmap showing the ChIP signal of STAT3 at RING1B-enriced loci upon inducing expression of STAT3-fused RYBP protein. (**G**) ChIP signal of RING1B at RING1B-enriced loci upon inducing expression of STAT3-fused RYBP protein, one-tailed Wilcoxon, n = 10165, data are presented as box plots showing the median (centre line), the 25th and 75th percentiles (box limits), and the minimum and maximum values (defined as the whisker ends, which extend to data points within 1.5 times the interquartile range from the box). (**H**) The volcano plot and venn diagram showing the expression changes of RYBP and RING1B-targeted genes following the expression of STAT3-fused RYBP. (**I**) GO analysis shows the pathways enriched by the upregulated RYBP and RING1B-targeted genes after the expression of STAT3-fused RYBP. (**J**) Venn diagram displays the number of upregulated and downregulated genes following RING1B deficiency. Data ref: GEO GSE132754, 2020. (**K**) Among the genes that were upregulated after RING1B deficiency, the volcano plot and venn diagram showing the quantity and proportion of

genes that were upregulated or downregulated following the expression of STAT3-fused RYBP. (**L**) The curve graph showing the signal distribution of STAT3 in RING1B-lacked regions. (**M**) The signal distribution of various factors in RING1B-lacked regions without STAT3 binding sites. (**N**) The heatmap showing the ChIP signal of each factor at RING1B-lacked and RING1B-enriched loci. Data ref: GEO GSE11431, 2008. (**O**) The percentage of STAT3 peaks overlapped with each factor. (**P, Q**) GO analysis demonstrates the biological processes involved in STAT3-targeted genes without RYBP and the genes co-targeted by RYBP and STAT3. (**R-T**) The heatmap and boxplot showing the ChIP signal of STAT3 at STAT3 with RYBP, or STAT3 without RYBP loci after RYBP depletion; n value is 1966 for Appendix Figure S2S and 397 for Appendix Figure S2T, data are presented as box plots showing the median (centre line), the 25th and 75th percentiles (box limits), and the minimum and maximum values (defined as the whisker ends, which extend to data points within 1.5 times the interquartile range from the box).

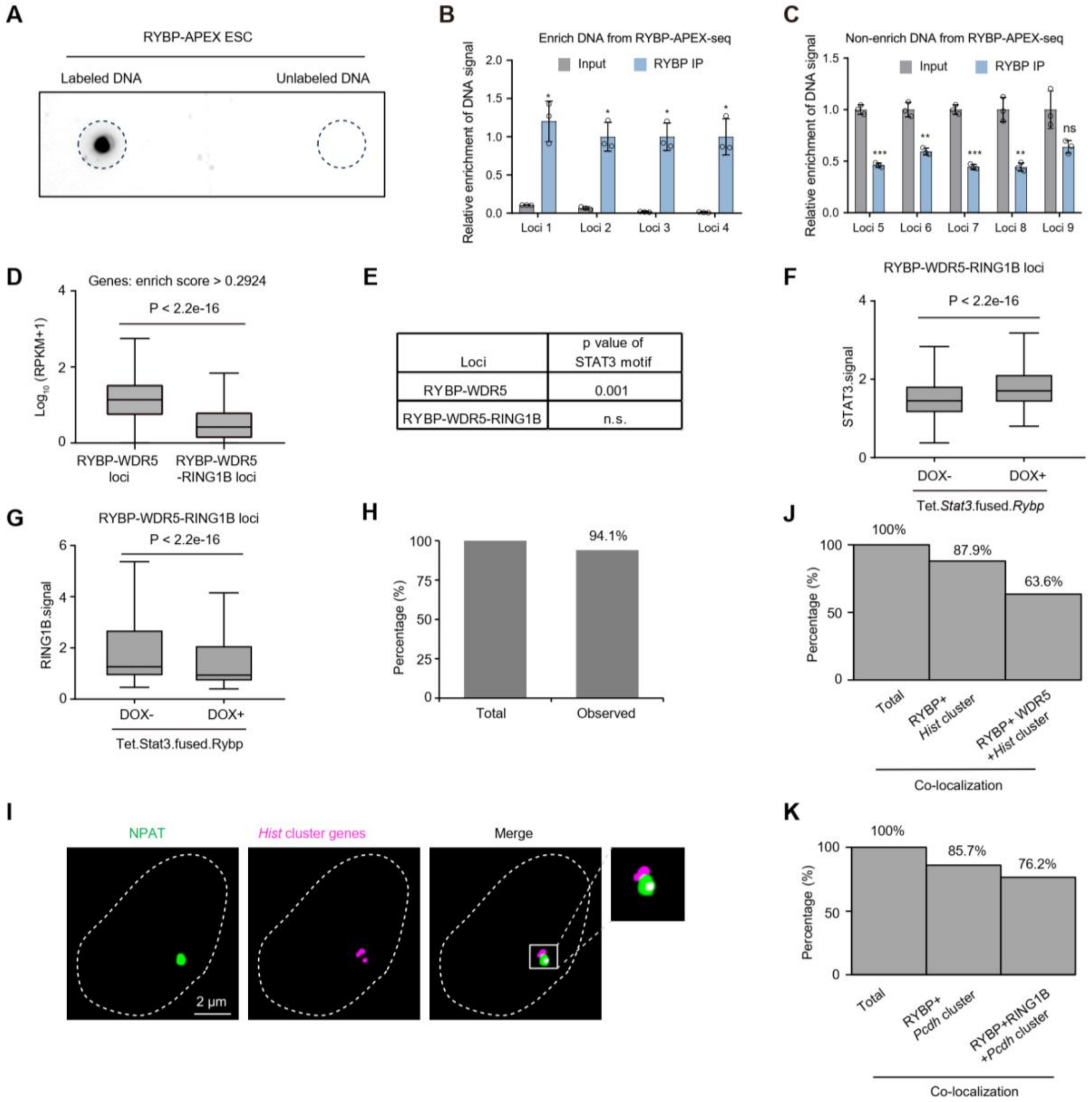

Appendix Figure S3. APEX-DNA-seq identifies RING1B-enriched and RING1B-lacked genes in RYBP condensates.

(**A**) DNA dot blotting showing the efficiency of APEX-mediated proximity labeling. (**B, C**) qPCR results showing the enrichment of DNA identified by RYBP APEX-DNA-seq with an enrichment score greater than 0.2924 (B), and the lower enrichment of DNA with an enrichment score less than 0.2924 (C). n = 3 for all groups, P values (from left to right) are 0.0188, 0.0126, 0.0107, 0.0185, 0.0005, 0.0035, 0.0003, 0.0088 and 0.0622, presented as the mean ± SD. (**D**) Boxplot showing the expression of genes at RYBP-WDR5 and RYBP-WDR5-RING1B loci, two-tailed Wilcoxon, n value are (from left to right) 4209 and 1177, data are presented as box plots showing the median (centre line), the

25th and 75th percentiles (box limits), and the minimum and maximum values (defined as the whisker ends, which extend to data points within 1.5 times the interquartile range from the box). (**E**) Motif analysis of STAT3 at RYBP-WDR5 loci and RYBP-WDR5-RING1B loci. (**F**, **G**) The enrichment of STAT3 (F) and RING1B (G) at RYBP-WDR5-RING1B loci upon the expression of RYBP-STAT3 fusion protein, one-tailed Wilcoxon, n value is 5642 for all the groups, data are presented as box plots showing the median (centre line), the 25th and 75th percentiles (box limits), and the minimum and maximum values (defined as the whisker ends, which extend to data points within 1.5 times the interquartile range from the box). (**H, I**) Percentage and 3D immuno-FISH images showing the co-localization between NPAT puncta and probe of *Hist* cluster genes. (**J**) Percentage of observed FISH signal of *Hist* cluster genes in RYBP or RYBP_WDR5 co-localized puncta. (**K**) Percentage of observed FISH signal of *Pcdh* cluster genes in RYBP or RYBP_RING1B co-localized puncta.

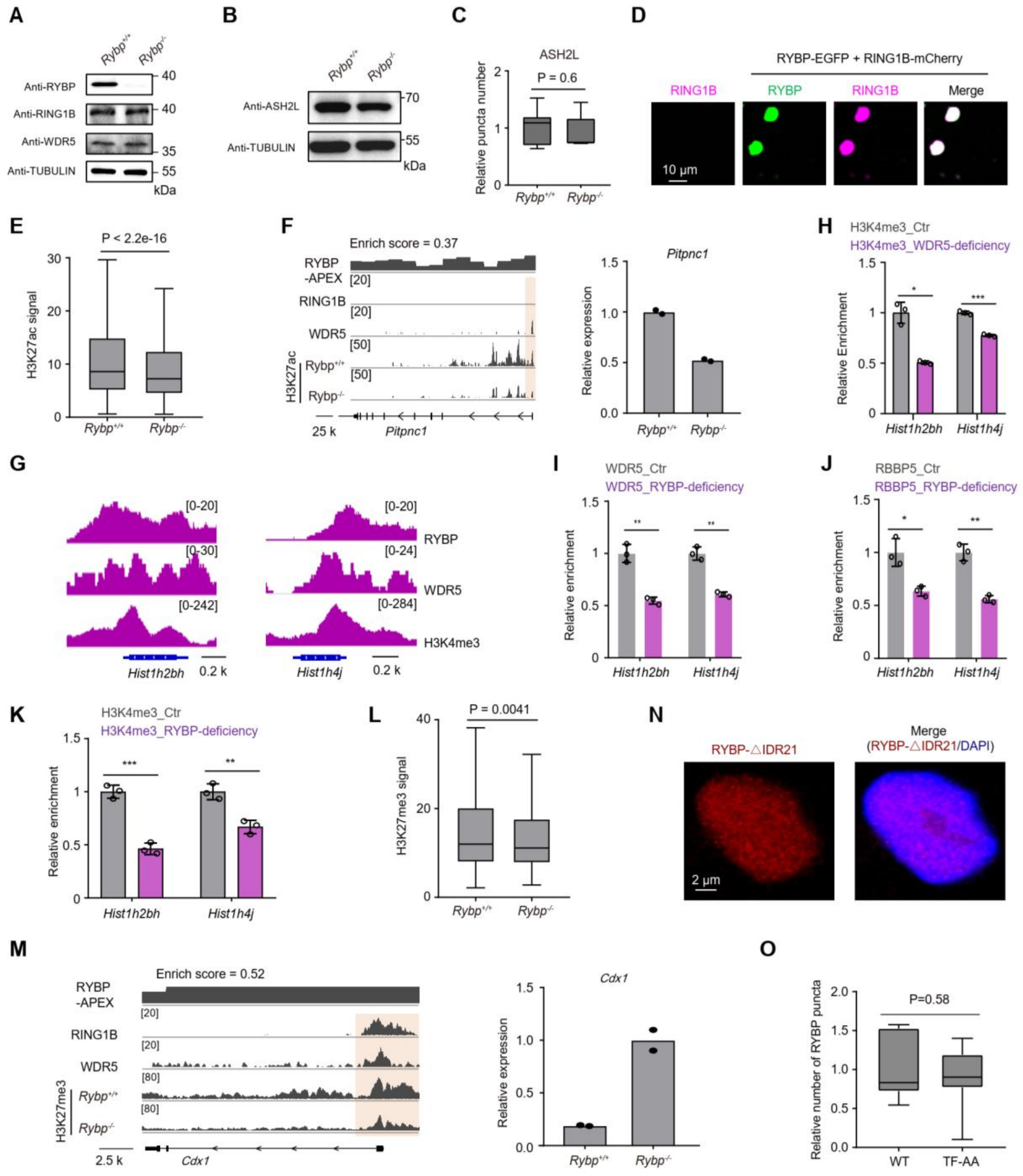

A
Rybp+/+
Rybp-/-
Anti-RYBP
Anti-RING1B
Anti-WDR5
Anti-TUBULIN
kDa
B
Anti-ASH2L
Anti-TUBULIN
C
ASH2L
P = 0.6
Relative puncta number
D
RYBP-EGFP + RING1B-mCherry
RING1B
RYBP
Merge
10 μm
E
P < 2.2e-16
H3K27ac signal
F
Enrich score = 0.37
RYBP -APEX
RING1B
WDR5
H3K27ac
Pitpnc1
Relative expression
H
H3K4me3_Ctr
H3K4me3_WDR5-deficiency
Relative Enrichment
Hist1h2bh
Hist1h4j
G
RYBP
WDR5
H3K4me3
I
WDR5_Ctr
WDR5_RYBP-deficiency
Relative enrichment
J
RBBP5_Ctr
RBBP5_RYBP-deficiency
K
H3K4me3_Ctr
H3K4me3_RYBP-deficiency
L
P = 0.0041
H3K27me3 signal
N
RYBP-△IDR21
Merge
(RYBP-△IDR21/DAPI)
2 μm
M
Enrich score = 0.52
H3K27me3
Cdx1
O
P=0.58
Relative number of RYBP puncta
WT
TF-AA

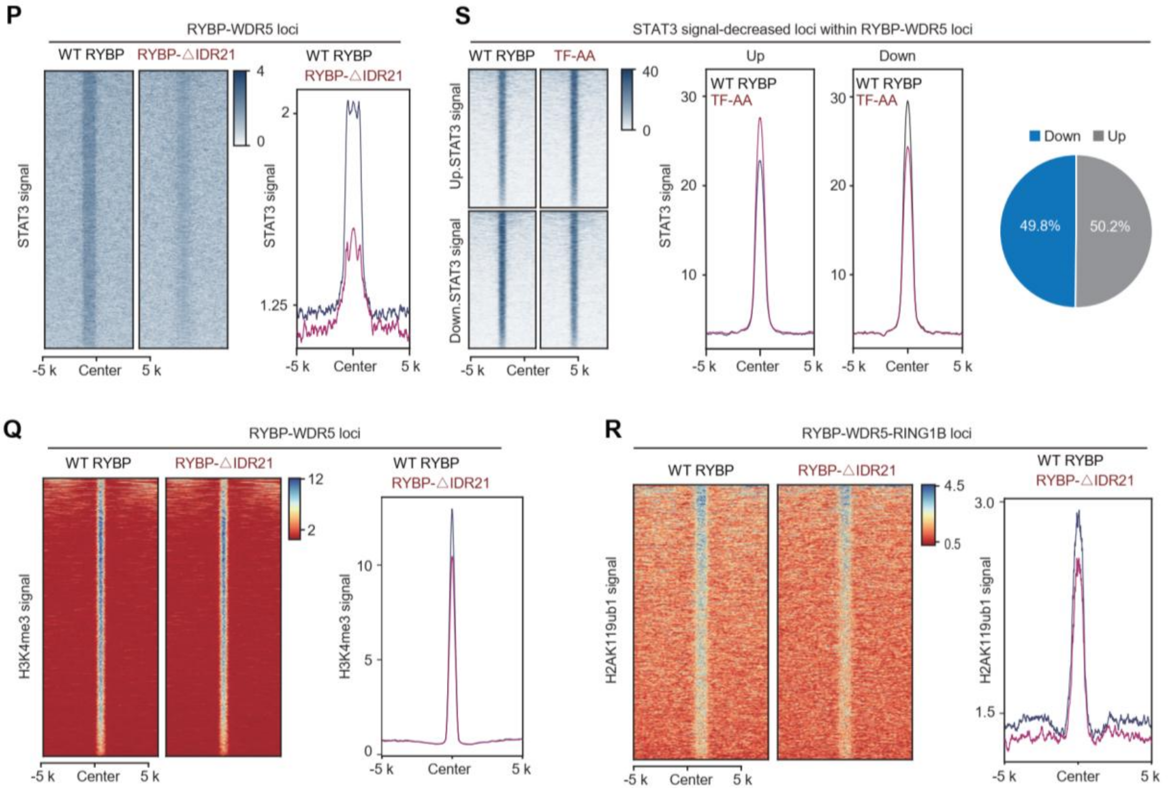

Appendix Figure S4. RYBP depletion reduces the genomic binding of RING1B and WDR5.

(**A, B**) Western blot showing expression change of RYBP, RING1B, WDR5 and ASH2L after RYBP depletion for 2 days. (**C**) The boxplot showing the quantity change of ASH2L puncta after RYBP depletion. Data are presented as box plots showing the median (centre line), the 25th and 75th percentiles (box limits), whisker ends are the minimum and maximum values. Two-tailed Welch's t-test; cell numbers (from left to right) are: n = 135, n = 72. (**D**) RYBP droplets incorporate PcG component (RING1B) protein *in vitro*. (**E**) ChIP signals of H3K27ac at the RYBP-WDR5 loci after RYBP depletion, n values are 5127 for the two groups, one-tailed Wilcoxon. Data are presented as box plots showing the median (centre line), the 25th and 75th percentiles (box limits), and the minimum and maximum values (defined as the whisker ends, which extend to data points within 1.5 times the interquartile range from the box). (**F**) ChIP-seq profiles of H3K27ac at *Pitinc1* locus and its expression before and after RYBP depletion, n = 2. (**G**) The signal of RYBP, WDR5 and H3K4me3 at the *Hist1h2bh* and *Hist1h4j* loci in WT ESCs. (**H**) ChIP-qPCR showing the enrichment changes of H3K4me3 at *Hist1h2bh* and *Hist1h4j* loci after WDR5-

deficiency. Two-tailed Welch's t-test; n = 3 for the two groups, P values (from left to right) are 0.0135 and 0.0002, presented as the mean ± SD. (**I-K**) ChIP-qPCR showing the enrichment changes of WDR5, RBBP5 and H3K4me3 at *Hist1h2bh* and *Hist1h4j* loci after RYBP-deficiency. Two-tailed Welch's t-test; n = 3 for all groups, P values (from left to right) are 0.0061, 0.004, 0.03, 0.0045, 0.0004 and 0.0045, presented as the mean ± SD. (**L**) ChIP signals of H3K27me3 after RYBP depletion at RYBP-WDR5-RING1B, n values are 1669 for the two groups, one-tailed Wilcoxon. Data are presented as box plots showing the median (centre line), the 25th and 75th percentiles (box limits), and the minimum and maximum values (defined as the whisker ends, which extend to data points within 1.5 times the interquartile range from the box). (**M**) ChIP-seq binding profiles of H3K27me3 at *Cdx1* locus and its expression before and after RYBP depletion, n = 2. Data ref: GEO GSE83135, 2016. (**N**) Representative images showing the nuclear localization of RYBP-△IDR21. (**O**) Relative number of WT RYBP and TF-AA RYBP puncta, cell number are (from left to right): n = 55 cells, n = 71 cells, Two-tailed Welch's t-test. Data are presented as box plots showing the median (centre line), whisker ends are the minimum and maximum values. (**P-R**) The heatmap and curve graph showing the ChIP signal of STAT3 (P), H3K4me3 (Q) and H2AK119ub1 (R) at numerous loci following the phase disruption of RYBP. (**S**) Among the STAT3 signal-decreased loci following RYBP phase disruption, the ratio of upregulation and downregulation of STAT3 signals following the TF-AA mutation. *P < 0.05; **P < 0.01, ***P < 0.001.

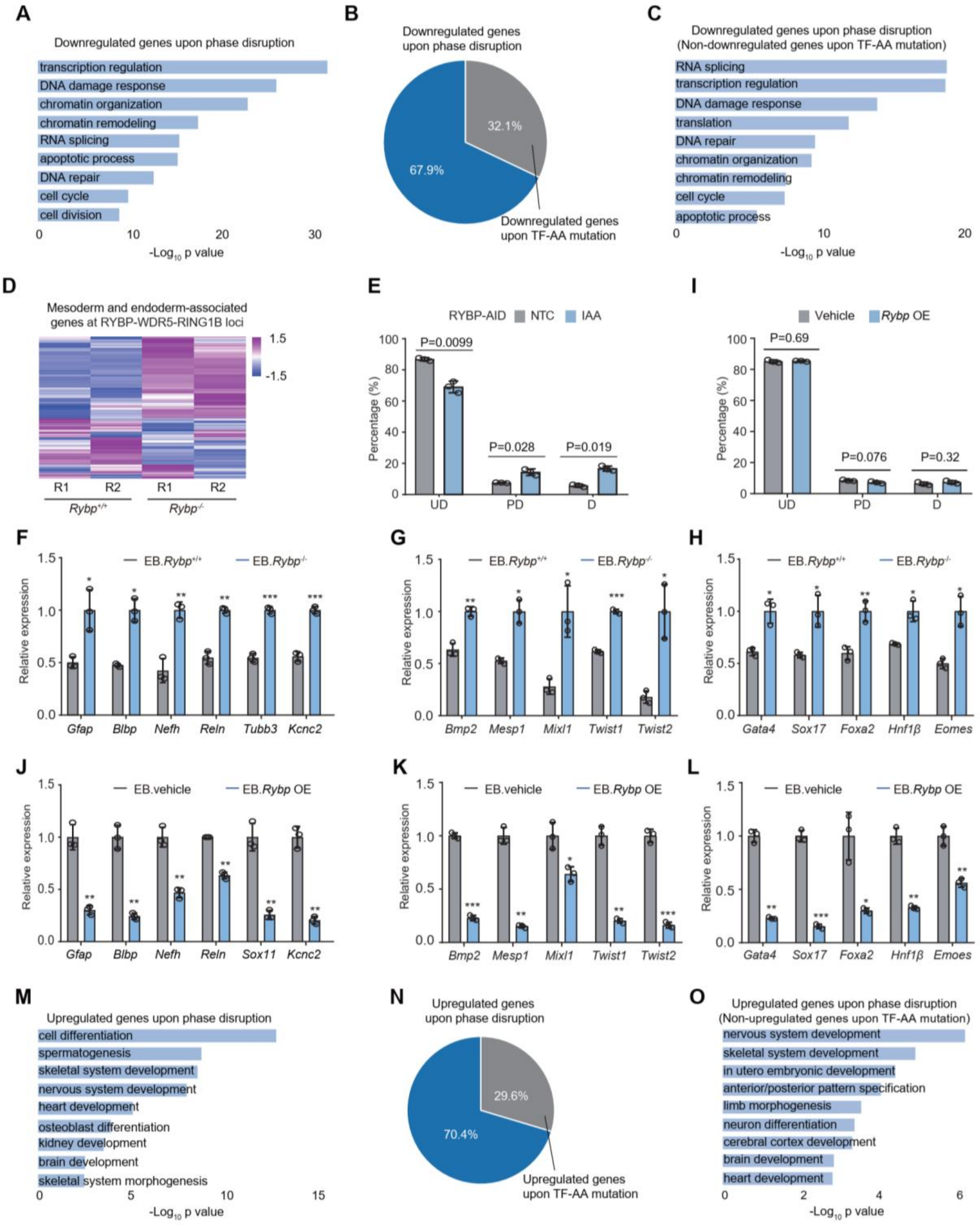

Appendix Figure S5. RYBP regulates the cell fate transition of ESCs.

(**A**) GO analysis showing the biological process of down-regulated genes following the RYBP phase disruption. (**B**) Among the downregulated genes following RYBP phase disruption, the ratio of downregulated genes following the TF-AA mutation. (**C**) GO

analysis showing the biological process of 67.9% non-downregulated genes in Appendix Figure S5B upon TF-AA mutation. (**D**) Heatmap showing the expression alteration of mesoderm and endoderm-associated genes at RYBP-WDR5-RING1B loci after RYBP depletion. (**E**) The differentiation ratio of ESC colonies after RYBP depletion, UD denotes undifferentiation, PD denotes partial differentiation, D denotes differentiation. Two-tailed Welch's t-test; n = 3 for all groups, presented as the mean ± SD. (**F-H**) RT-qPCR results indicate the expression levels of genes related to the ectoderm (F), mesoderm (G), and endoderm (H) in EB samples following RYBP knockout. Two-tailed Welch's t-test; n = 3 for all groups, P values (from left to right) are 0.04, 0.012, 0.0032, 0.0015, 0.00014, 0.00024, 0.0017, 0.015, 0.028, 0.000021, 0.027, 0.02, 0.038, 0.0068, 0.031 and 0.0189, presented as the mean ± SD. (**I**) The differentiation ratio of ESC colonies after RYBP overexpression. Two-tailed Welch's t-test; n = 3 for all groups, presented as the mean ± SD. (**J-L**) RT-qPCR results indicate the expression levels of genes related to the ectoderm (J), mesoderm (K), and endoderm (L) in EB samples following RYBP overexpression. Two-tailed Welch's t-test; n = 3 for all groups, P values (from left to right) are 0.0056, 0.0048, 0.0035, 0.0027, 0.0063, 0.0024, 4.47e-06, 0.0021, 0.0208, 0.0027, 0.0003, 0.0014, 0.0003, 0.0313, 0.0028 and 0.0061, presented as the mean ± SD. (**M**) GO analysis showing the biological process of up-regulated genes following RYBP phase disruption. (**N**) Among the up-regulated genes following RYBP phase disruption, the ratio of up-regulated genes following the TF-AA mutation. (**O**) GO analysis showing the biological process of 70.4% non-upregulated genes in Appendix Figure S5N upon TF-AA mutation. *P < 0.05; **P < 0.01, ***P < 0.001.

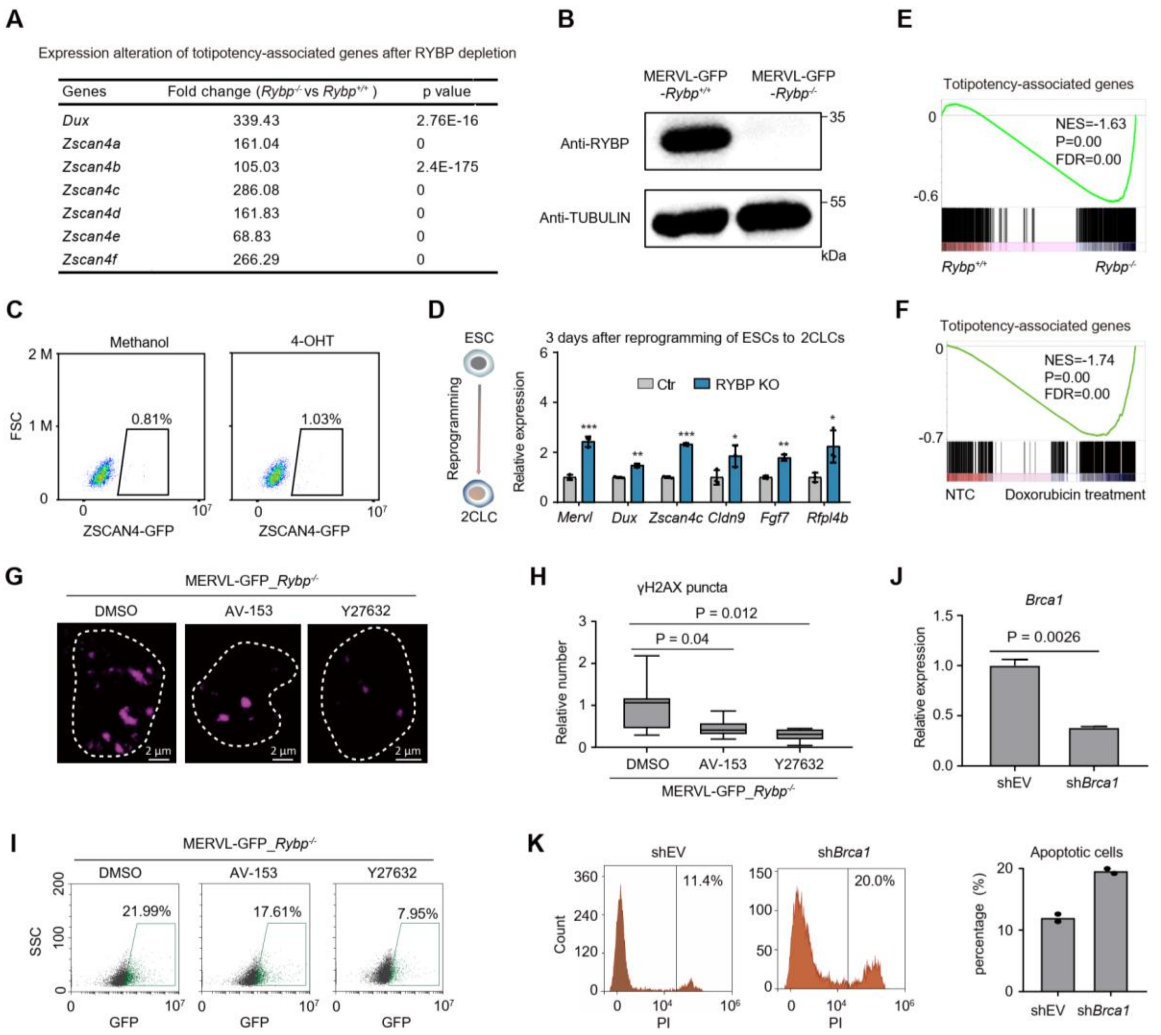

Appendix Figure S6. RYBP depletion-induced DNA damage facilitates ESC-to-2CLC transition.

(**A**) A table summarizes the expression changes of typical totipotency-associated genes after RYBP knockout. (**B**) Western blot showing the deficiency of RYBP in MERVL-GFP_*Rybp*$^{+/+}$ cells with the treatment of 4-OHT for 4 days. (**C**) Flow cytometry showing the change in the proportion of GFP-positive cells in ZSCAN4-GFP cells after 4 days of 4-OHT treatment. (**D**) Left: a model showing the reprogramming process from ESCs to 2CLCs. Right: relative mRNA expression of totipotency-related genes after RYBP depletion for 3 days after reprogramming from ESCs to 2CLCs. One-tailed Welch's t-test; n = 3 for the two groups. P values (from left to right) are 0.00085, 0.0019, 6.7e-6, 0.028, 0.0016 and 0.035, presented as the mean ± SD. (**E**) Gene set enrichment analysis of the RNA-seq data after RYBP depletion, totipotency-related genes are used. (**F**) Gene set

enrichment analysis of the RNA-seq data from non-Doxorubicin and Doxorubicin treated ESCs, totipotency-related genes are used. (**G**, **H**) Representative images and histogram showing the puncta number of γH2AX in different groups, cell number are (from left to right): n = 44 cells, n = 74 cells, n = 81 cells, data are presented as box plots showing the median (centre line), the 25th and 75th percentiles (box limits), whisker ends are the minimum and maximum values. (**I**) Flow cytometry showed that the percentage of MERVL-GFP positive cells in different groups. (**J**) RT-qPCR showing the efficiency of BRCA1 knockdown. Two-tailed Welch's t-test; n = 3 for the two groups, presented as the mean ± SD. (**K**) Flow cytometry and histogram showed that the percentage of apoptotic cells after *Brca1* knockdown, n = 2.

Appendix Table S1. Information of FISH probes

Probe ID	chromosome	Start position	End position
probe 1	chr5	120913667	120917333
probe 2	chr5	135579407	135580835
probe 3	chr17	56443734	56462908
probe 4	chr17	56092045	56096445
probe 5	chr17	17511294	17531862
probe 6	chr8	74414504	74464723
probe 7	chr7	112024808	112038041
probe 8	chr7	112176480	112227231

Appendix Table S2. Primers used for RT-qPCR analysis

Name	Orientation	Sequence
Nefh	F	GTTCCGAGTGAGGTTGGACC
	R	CCGCCGGTACTCAGTTATCTC
kcnc2	F	TCGCCACCCAGGAGTATTC
	R	CTCCACGTCGGTCTCATCG
Sox17	F	CGCACGGAATTCGAACAGTA
	R	GTCAAATGTCGGGGTAGTTG
Mixl1	F	ATCCGCCCGGACCCTCCAAA
	R	TCGGTTCTGGAACCACACCTGGA
Sfrp1	F	TACTGGCCCGAGATGCTCAA
	R	GAGGCTTCCGTGGTATTGGG
Ezh2	F	AGCACAAGTCATCCCGTTAAAG
	R	AATTCTGTTGTAAGGGCGACC
Pcgf6	F	GAGGAGCGCCTGATAAACCTT
	R	ACACTCTGTAATGGTGGTTGC
Zscan4c	F	CCGGAGAAAGCAGTGAGGTGGA
	R	CGAAAATGCTAACAGTTGAT
Cldn9	F	GTTGTGGCCCAAGTGGTATG
	R	GCGGTGAGTACGATACGGG
Fgf7	F	TGGGCACTATATCTCTAGCTTGC
	R	GGGTGCGACAGAACAGTCT
Rfpl4b	F	TGCCTGGACATTTACTCTCATCC
	R	CGGGACAGGTCATTATCAAGTC
