# Supplementary figures and images for: RYBP regulates selective genomic binding of TrxG and PcG components in embryonic stem cell fate control
Source: EMBO J. 2026 Apr 28;45(11):3808–32. doi: 10.1038/s44318-026-00788-y (PMC13226663; doi:10.1038/s44318-026-00788-y)

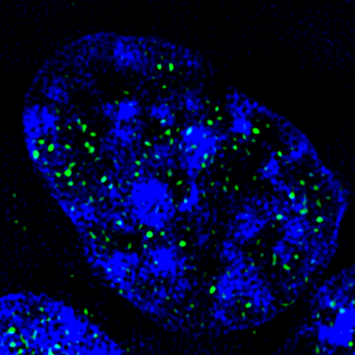

Supplement: Supplementary file 3 — Source data Fig. 1 [file 44318_2026_788_MOESM3_ESM.zip › Figure 1/Figure 1C/merge.tif]

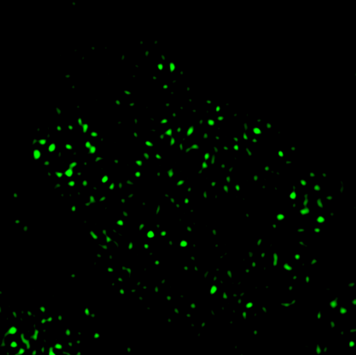

Supplement: Supplementary file 3 — Source data Fig. 1 [file 44318_2026_788_MOESM3_ESM.zip › Figure 1/Figure 1C/RYBP.tif]

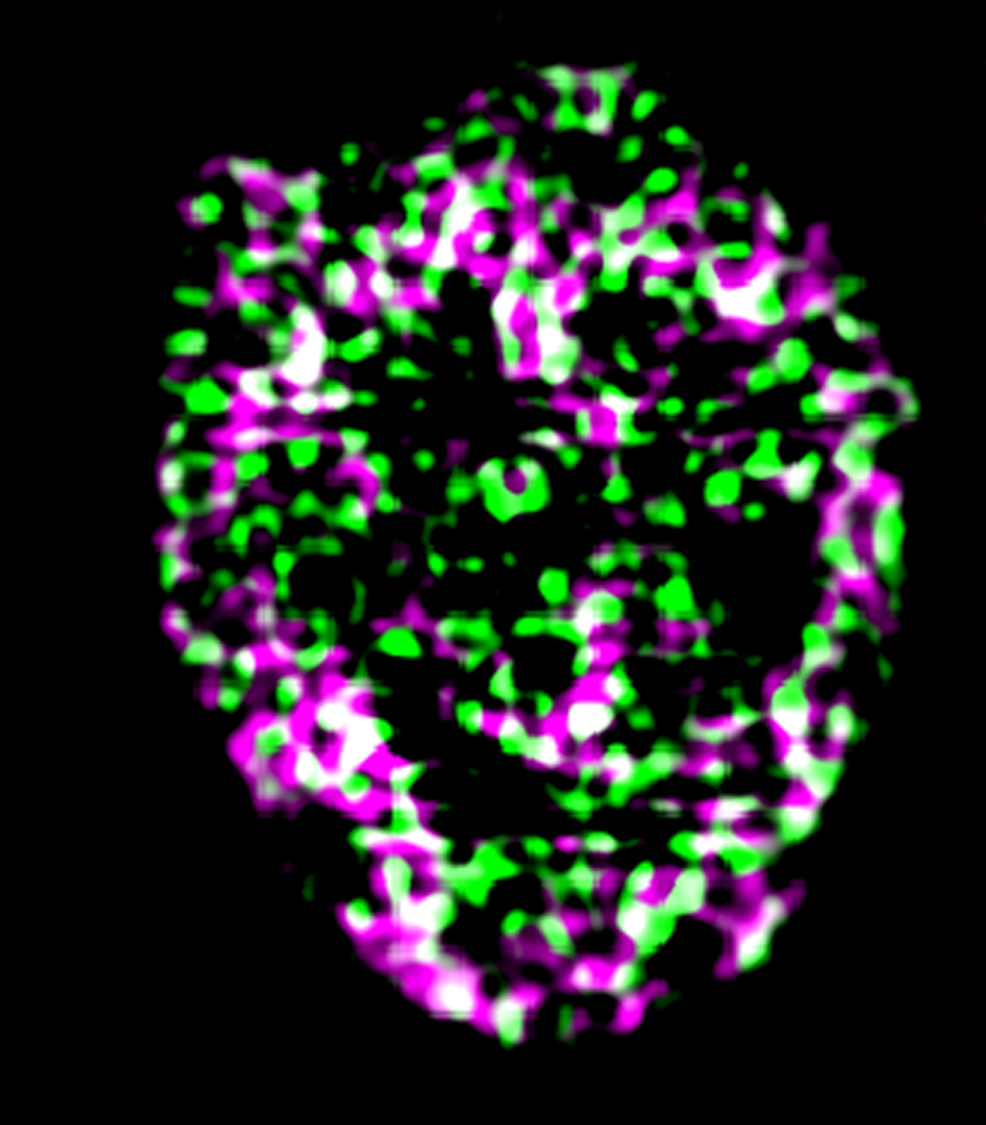

Supplement: Supplementary file 3 — Source data Fig. 1 [file 44318_2026_788_MOESM3_ESM.zip › Figure 1/Figure 1D/DPY30.merge.tif]

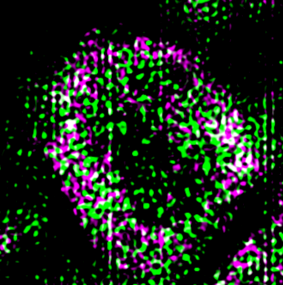

Supplement: Supplementary file 3 — Source data Fig. 1 [file 44318_2026_788_MOESM3_ESM.zip › Figure 1/Figure 1D/H2AK119ub1.merge.tif]

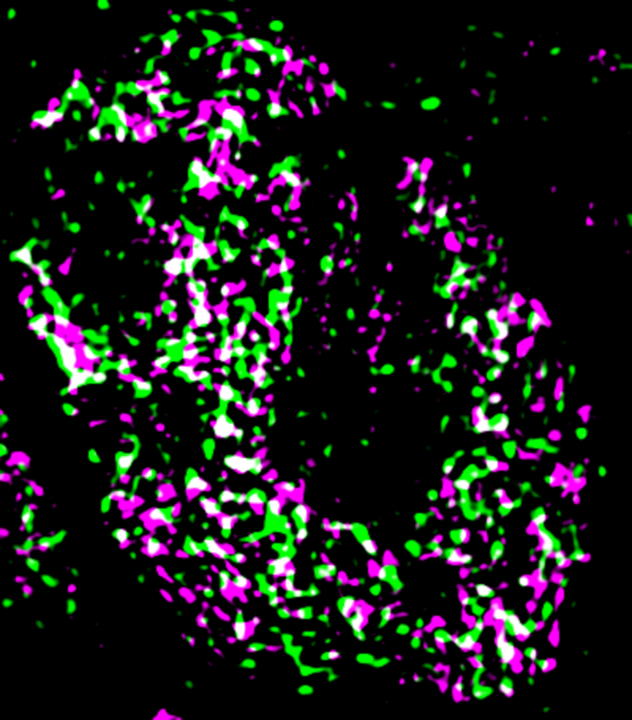

Supplement: Supplementary file 3 — Source data Fig. 1 [file 44318_2026_788_MOESM3_ESM.zip › Figure 1/Figure 1D/RING1B.merge.tif]

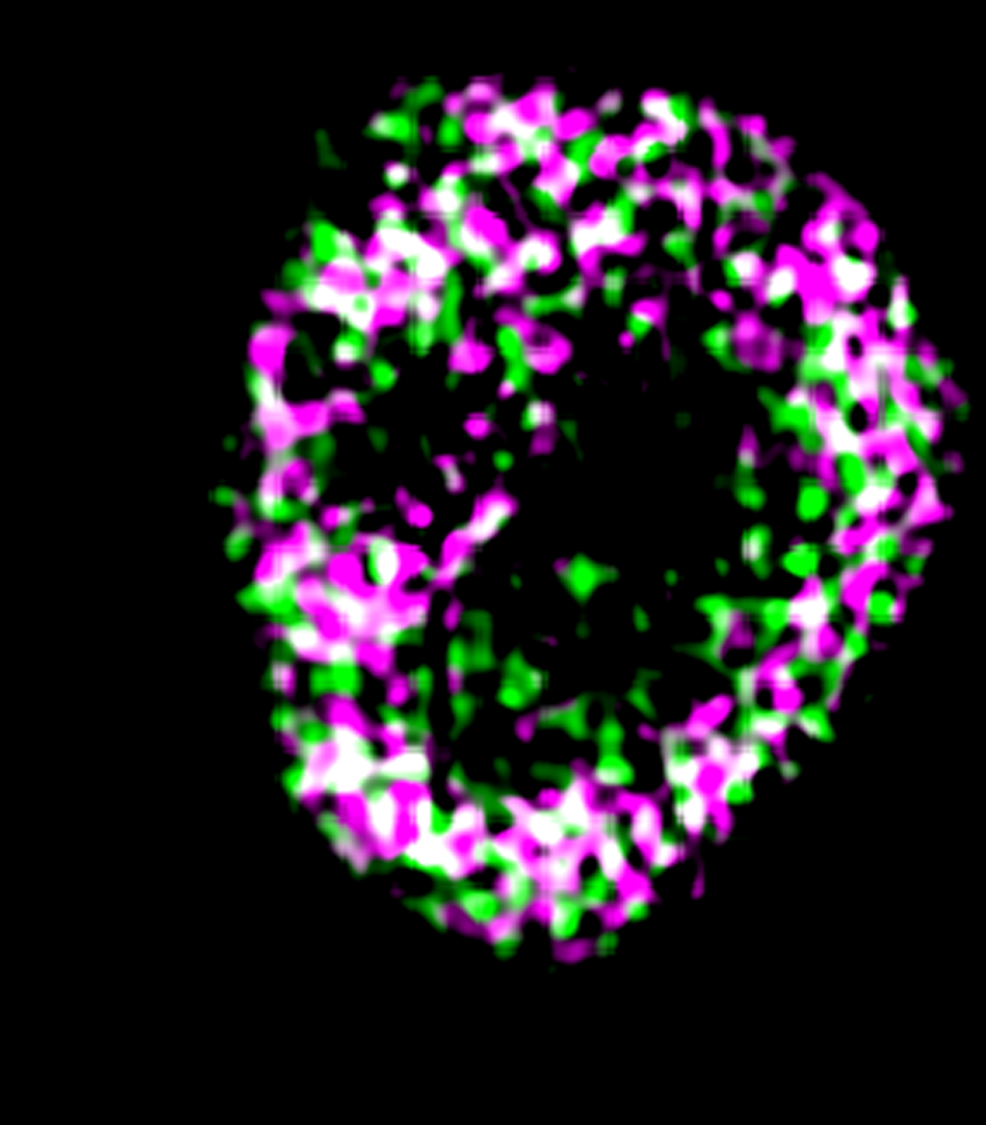

Supplement: Supplementary file 3 — Source data Fig. 1 [file 44318_2026_788_MOESM3_ESM.zip › Figure 1/Figure 1D/WDR5.merge.tif]

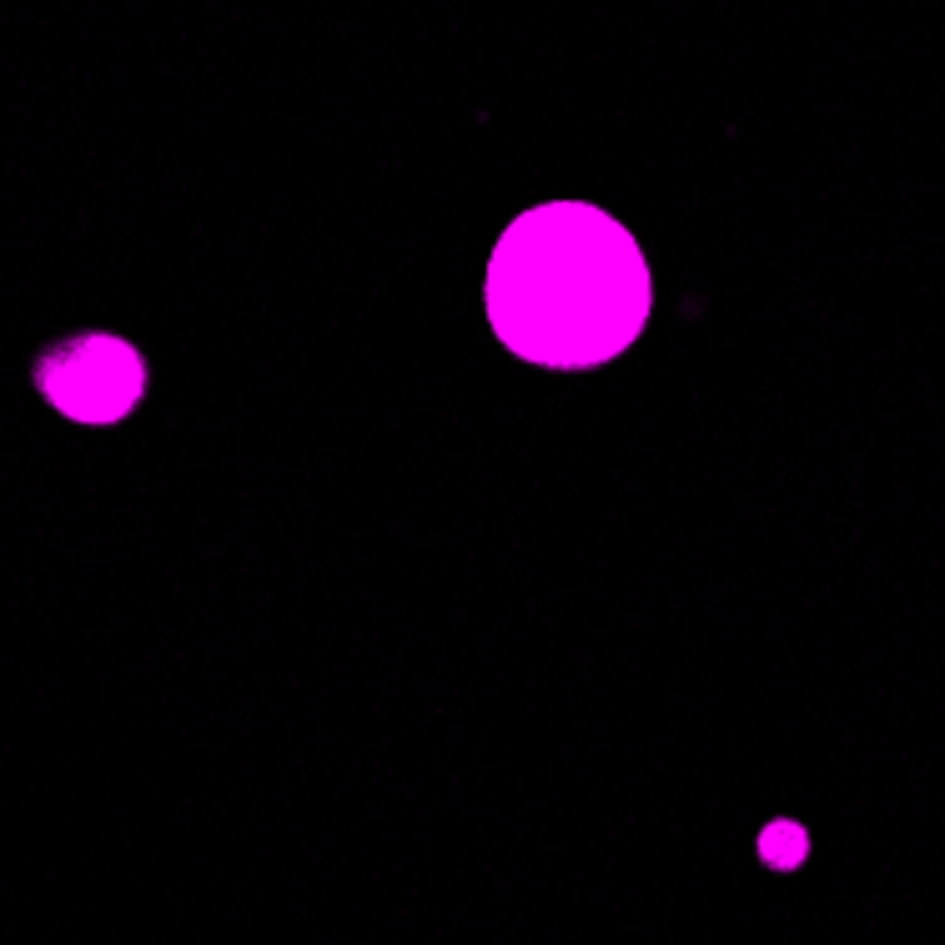

Supplement: Supplementary file 3 — Source data Fig. 1 [file 44318_2026_788_MOESM3_ESM.zip › Figure 1/Figure 1E/DPY30/DPY30.tif]

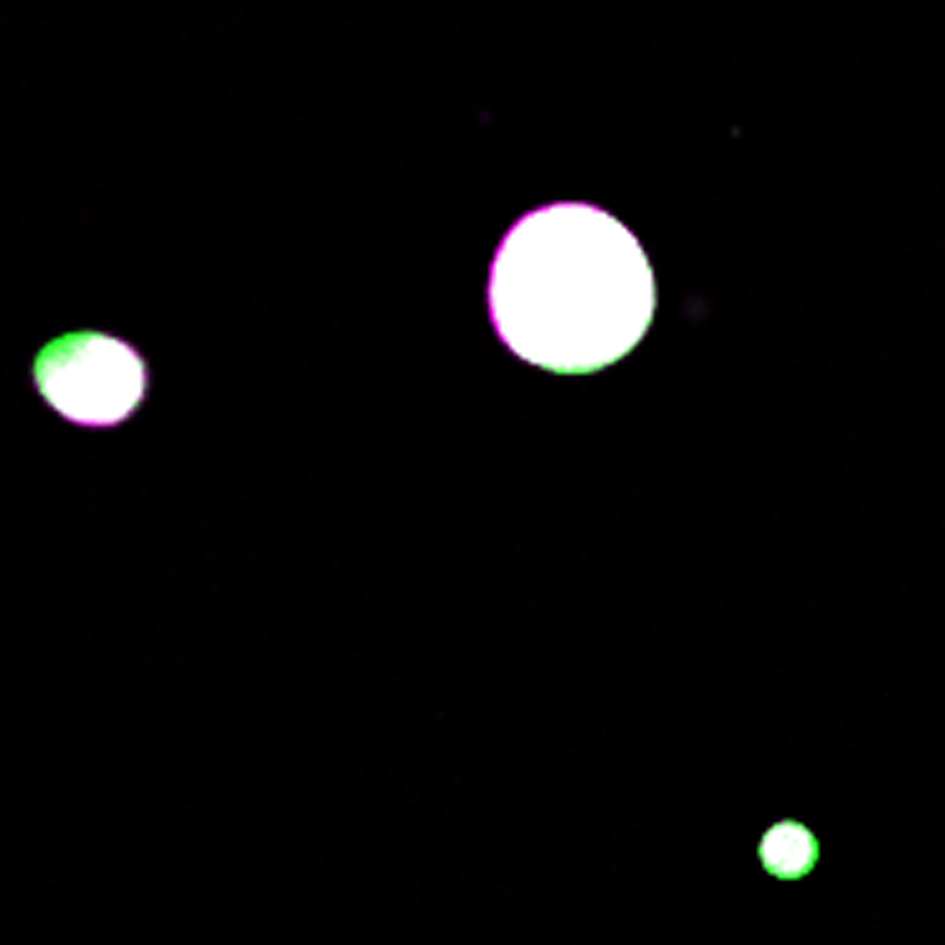

Supplement: Supplementary file 3 — Source data Fig. 1 [file 44318_2026_788_MOESM3_ESM.zip › Figure 1/Figure 1E/DPY30/merge.tif]

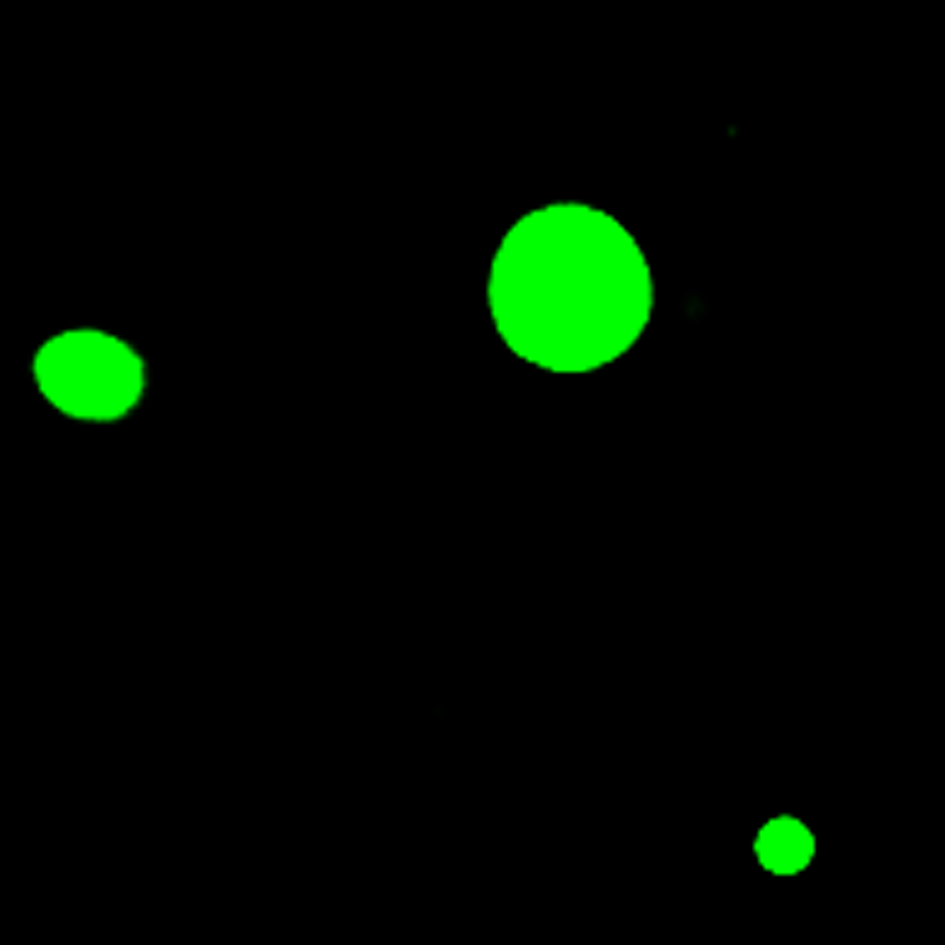

Supplement: Supplementary file 3 — Source data Fig. 1 [file 44318_2026_788_MOESM3_ESM.zip › Figure 1/Figure 1E/DPY30/RYBP.tif]

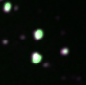

Supplement: Supplementary file 3 — Source data Fig. 1 [file 44318_2026_788_MOESM3_ESM.zip › Figure 1/Figure 1E/RING1B/merge.tif]

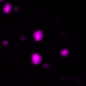

Supplement: Supplementary file 3 — Source data Fig. 1 [file 44318_2026_788_MOESM3_ESM.zip › Figure 1/Figure 1E/RING1B/ring1b.tif]

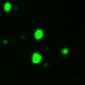

Supplement: Supplementary file 3 — Source data Fig. 1 [file 44318_2026_788_MOESM3_ESM.zip › Figure 1/Figure 1E/RING1B/rybp.tif]

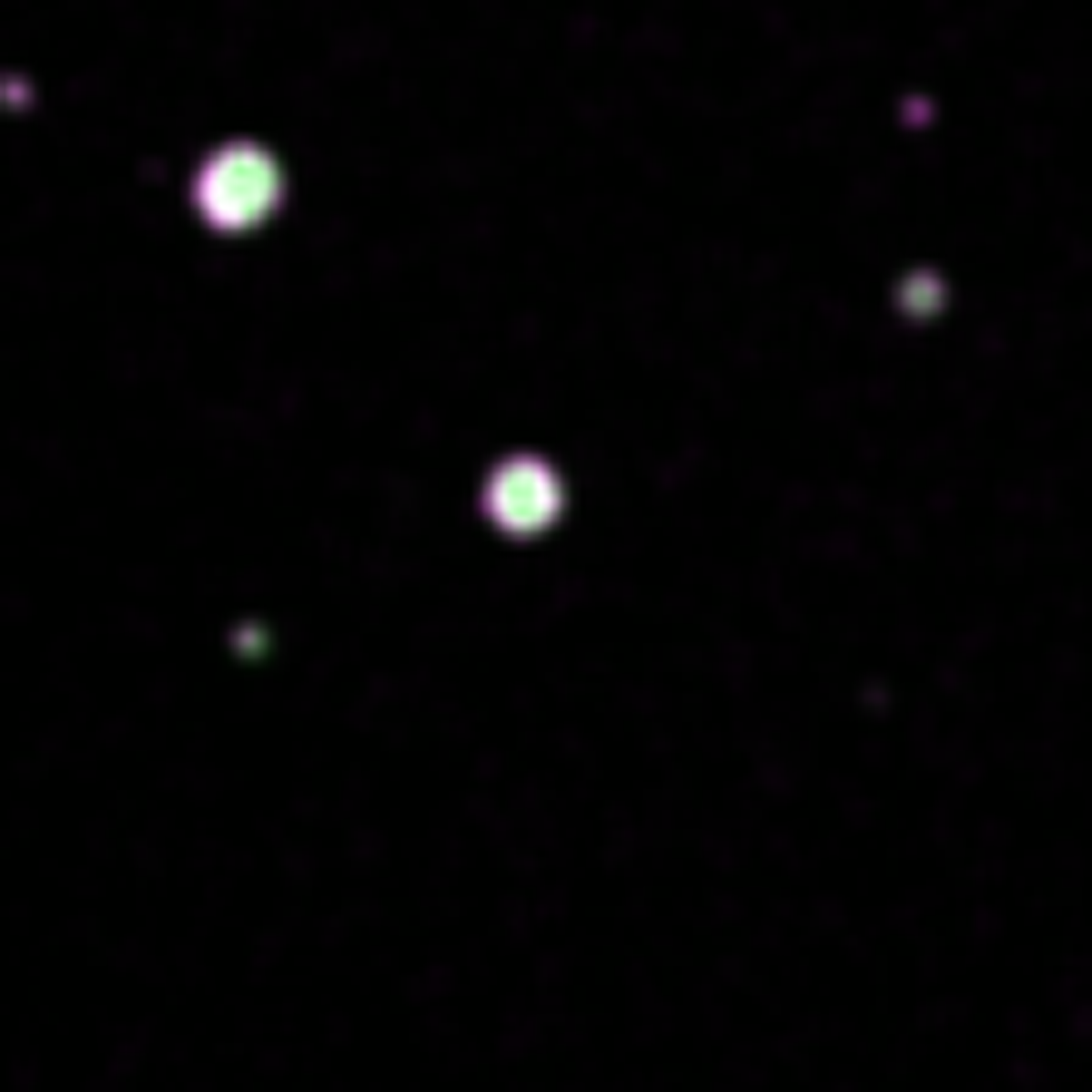

Supplement: Supplementary file 3 — Source data Fig. 1 [file 44318_2026_788_MOESM3_ESM.zip › Figure 1/Figure 1E/WDR5/merge.tif]

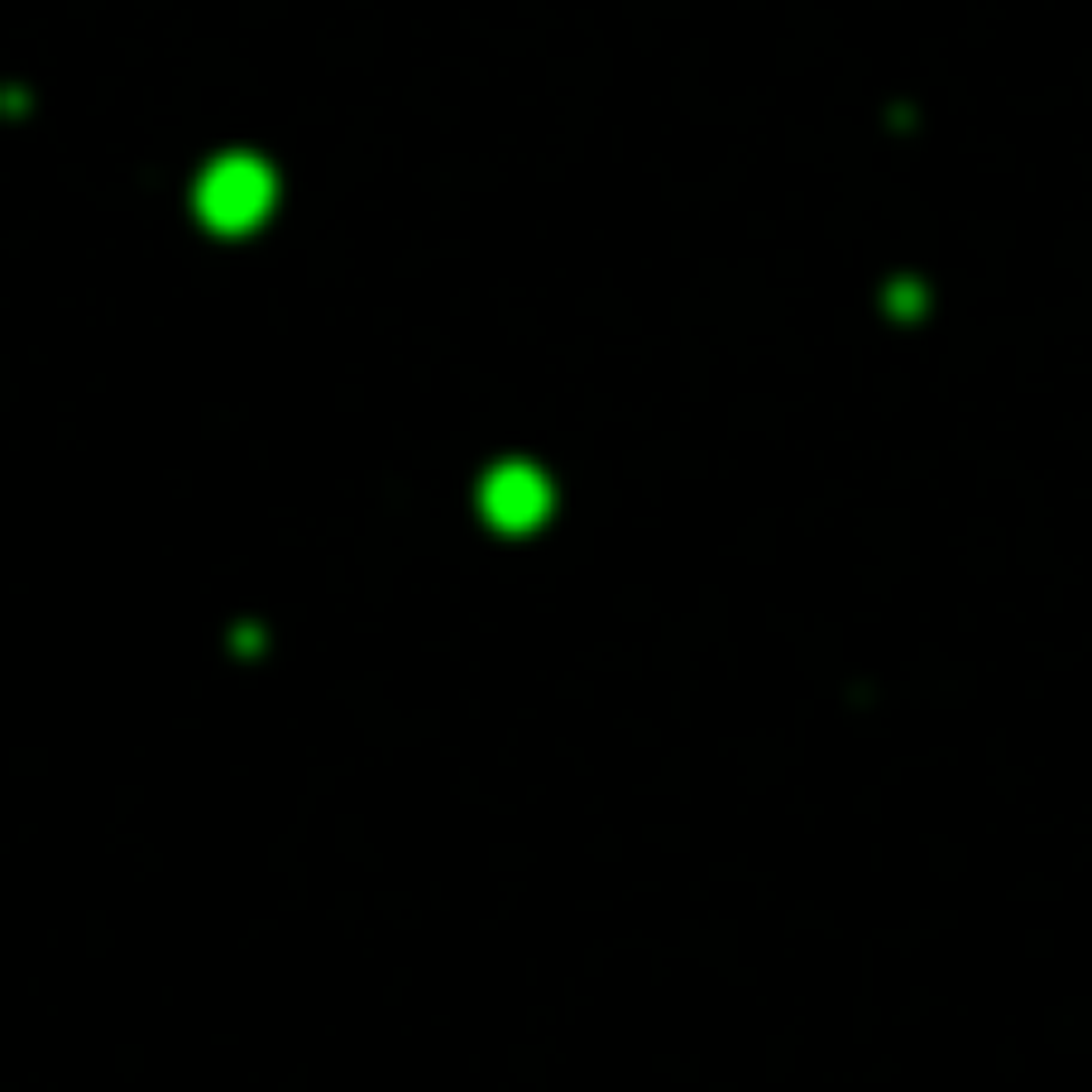

Supplement: Supplementary file 3 — Source data Fig. 1 [file 44318_2026_788_MOESM3_ESM.zip › Figure 1/Figure 1E/WDR5/RYBP.tif]

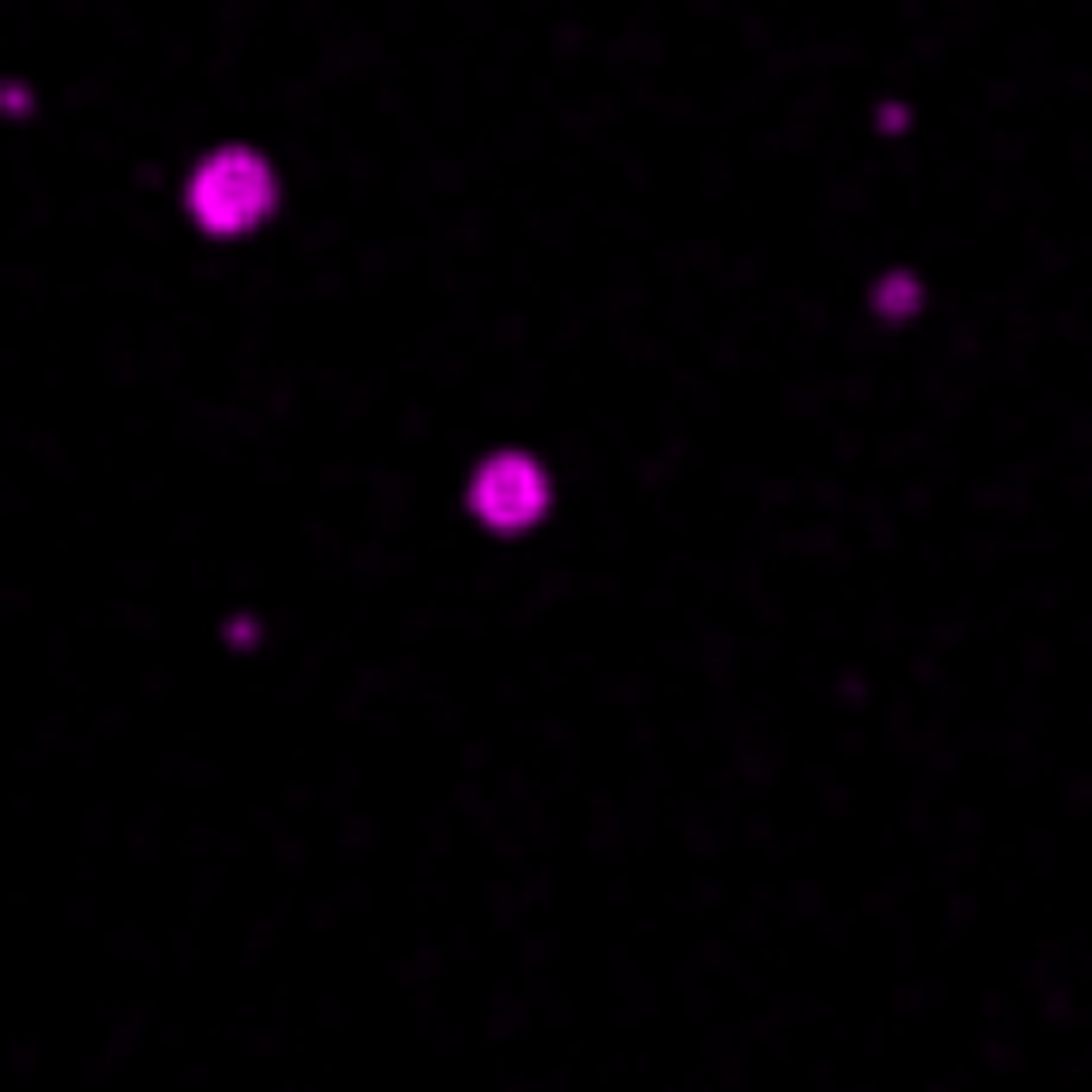

Supplement: Supplementary file 3 — Source data Fig. 1 [file 44318_2026_788_MOESM3_ESM.zip › Figure 1/Figure 1E/WDR5/WDR5.tif]

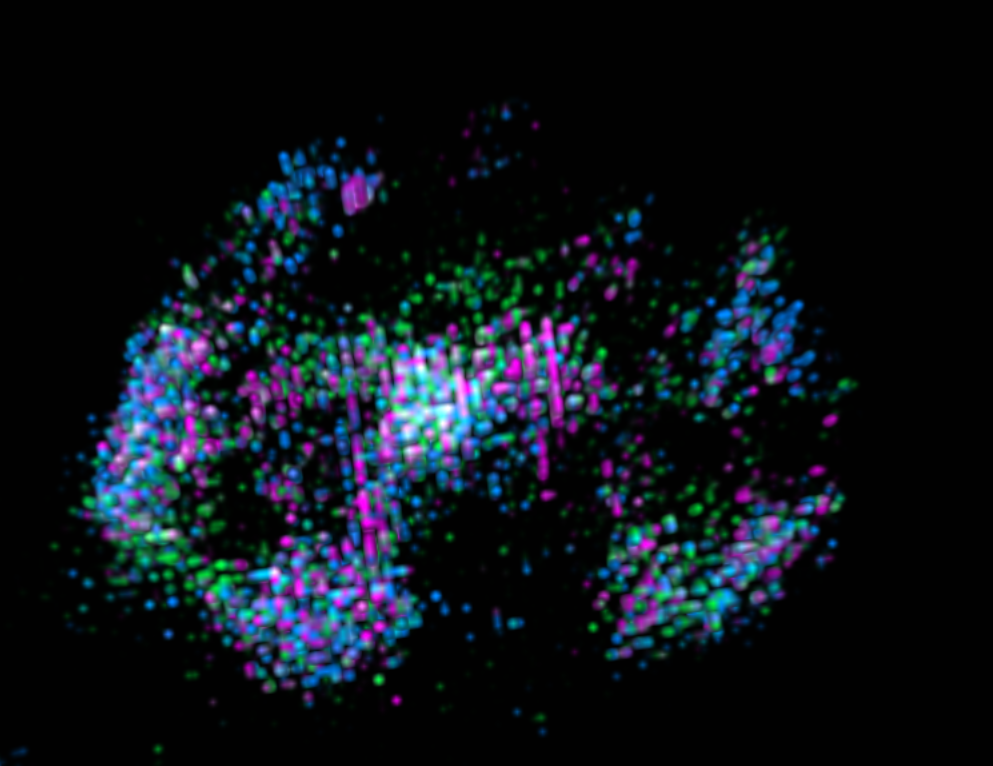

Supplement: Supplementary file 3 — Source data Fig. 1 [file 44318_2026_788_MOESM3_ESM.zip › Figure 1/Figure 1J/MERGE-cai.tif]

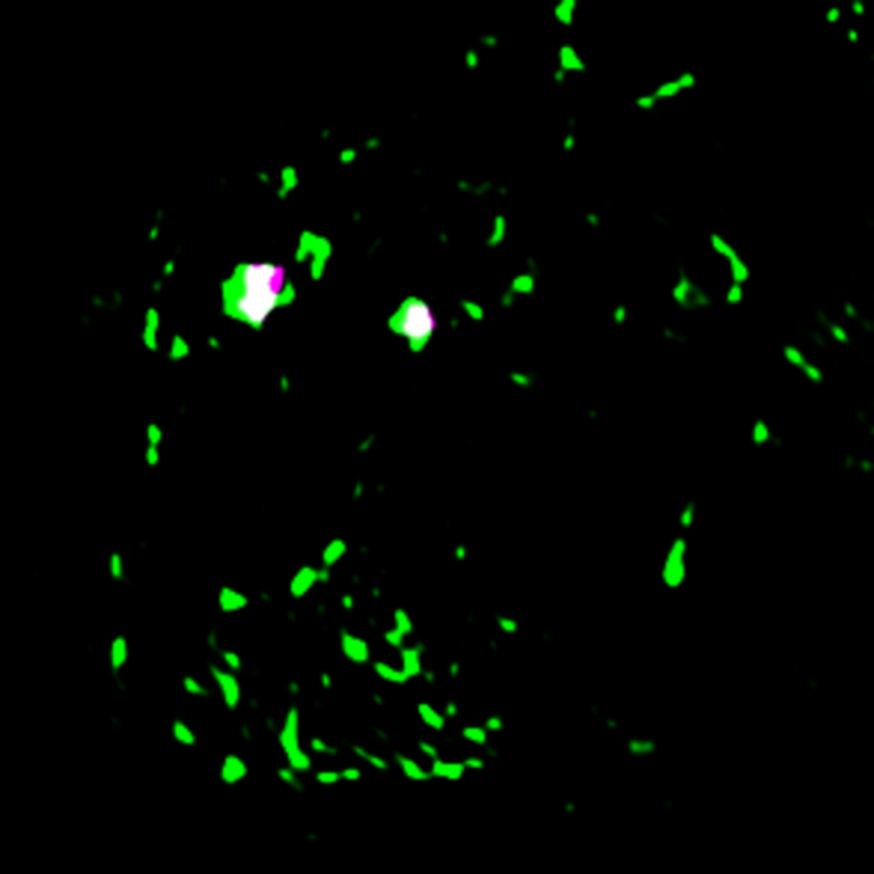

Supplement: Supplementary file 3 — Source data Fig. 1 [file 44318_2026_788_MOESM3_ESM.zip › Figure 1/Figure 1K/MERGE.NPAT+WDR5.tif]

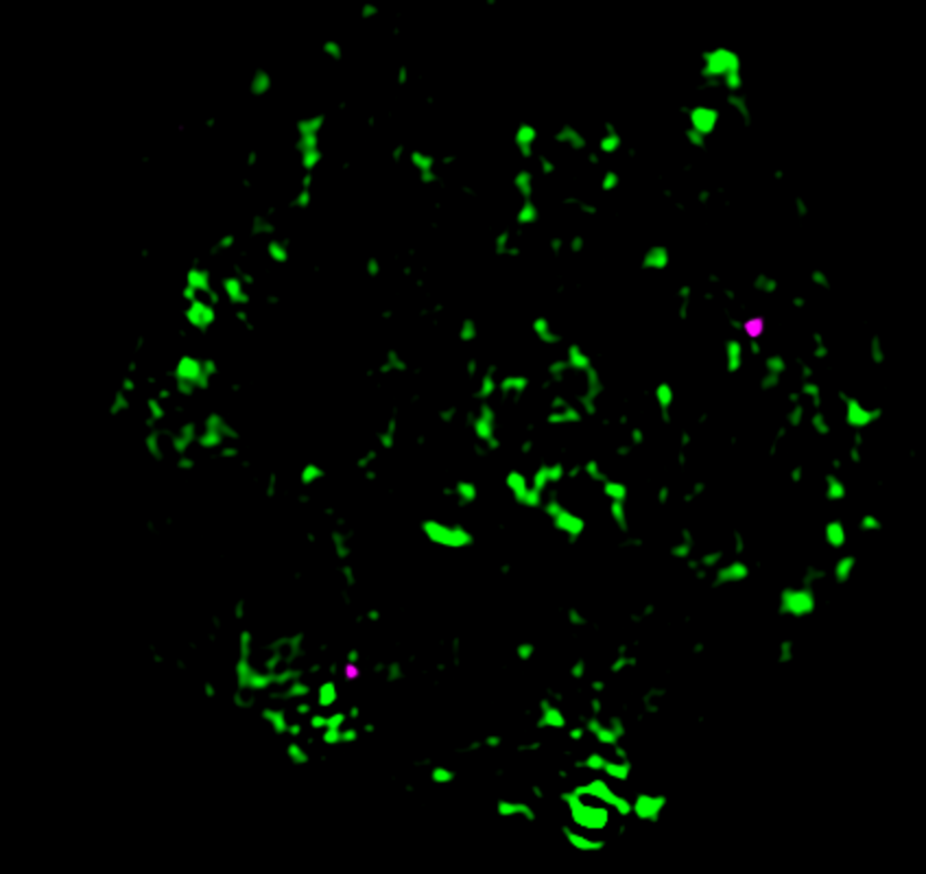

Supplement: Supplementary file 3 — Source data Fig. 1 [file 44318_2026_788_MOESM3_ESM.zip › Figure 1/Figure 1K/merge.RING1B+NPAT.tif]

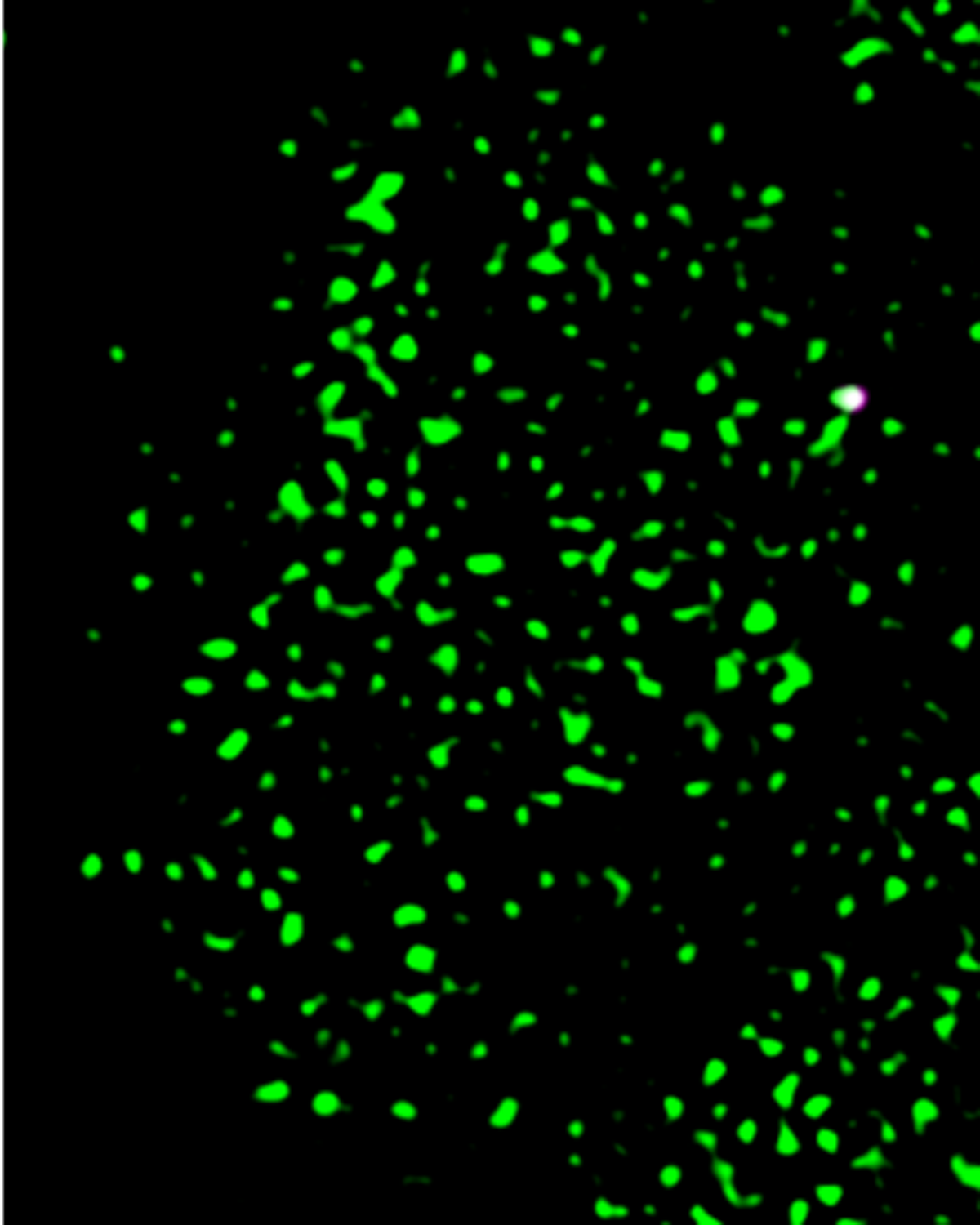

Supplement: Supplementary file 3 — Source data Fig. 1 [file 44318_2026_788_MOESM3_ESM.zip › Figure 1/Figure 1K/merge.RYBP+NPAT.tif]

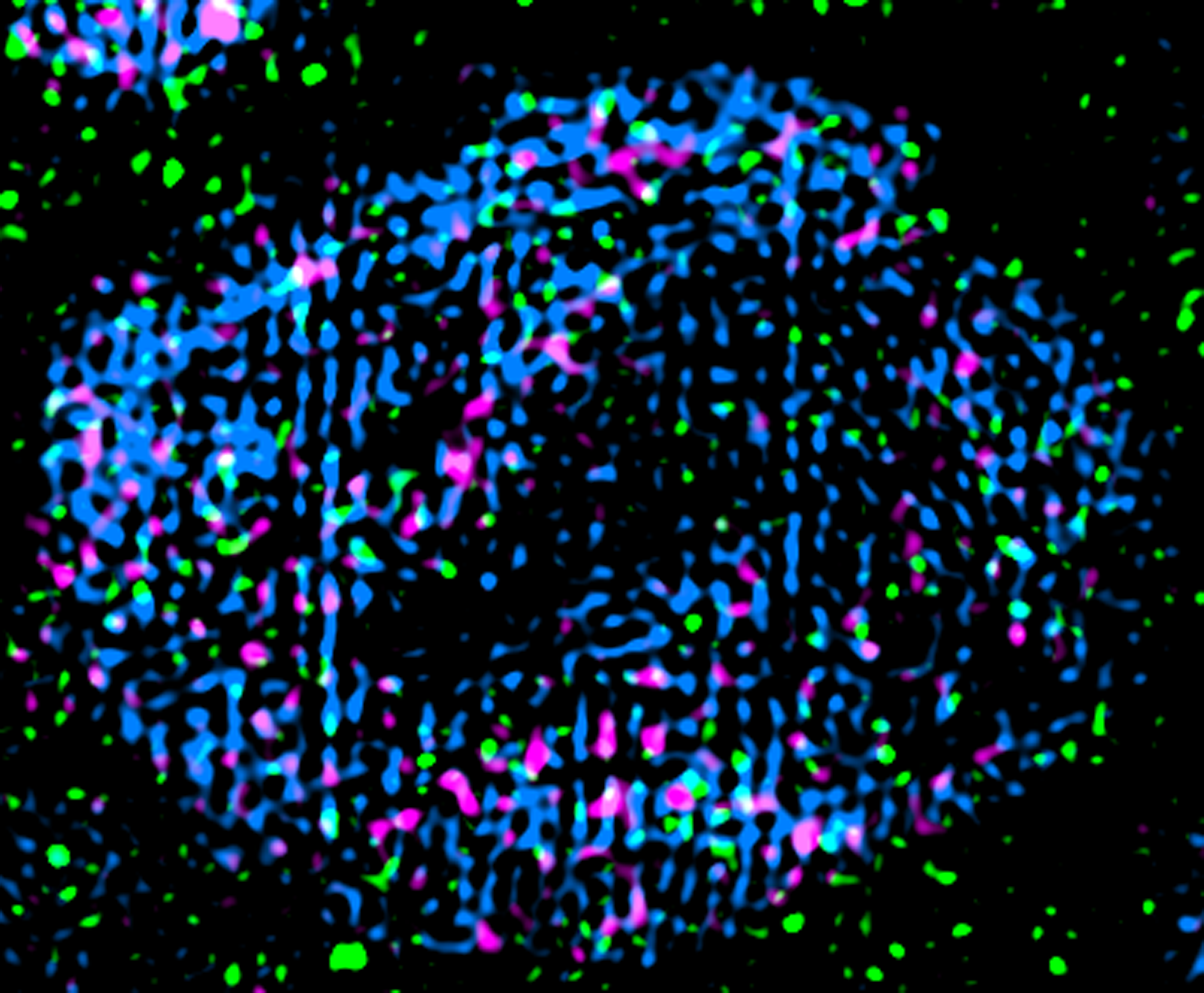

Supplement: Supplementary file 4 — Source data Fig. 2 [file 44318_2026_788_MOESM4_ESM.zip › Figure 2/Figure 2D,2F/Figure 2D.tif]

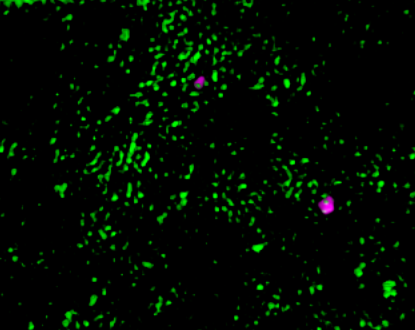

Supplement: Supplementary file 4 — Source data Fig. 2 [file 44318_2026_788_MOESM4_ESM.zip › Figure 2/Figure 2D,2F/Figure 2F.tif]

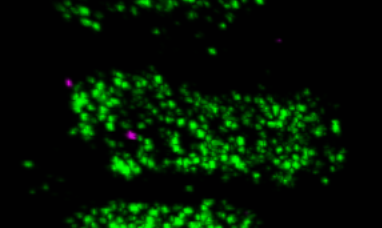

Supplement: Supplementary file 5 — Source data Fig. 3 [file 44318_2026_788_MOESM5_ESM.zip › Figure 3/Figure 3B/ES-0.57.tif]

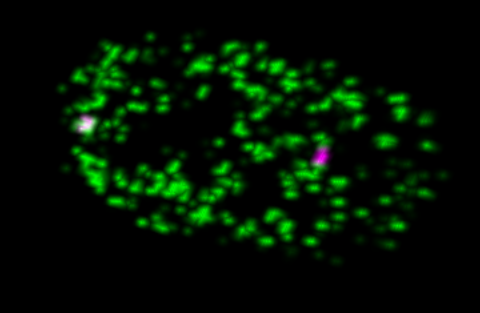

Supplement: Supplementary file 5 — Source data Fig. 3 [file 44318_2026_788_MOESM5_ESM.zip › Figure 3/Figure 3B/ES0.87.tif]

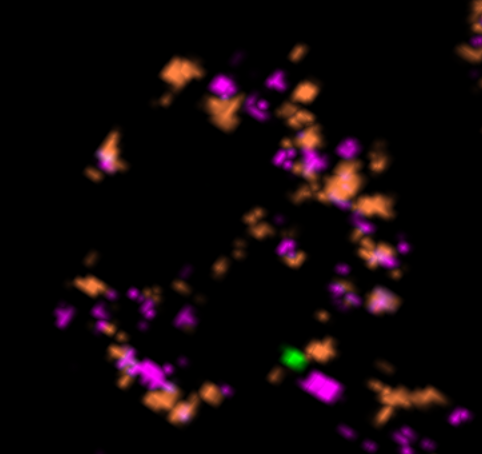

Supplement: Supplementary file 5 — Source data Fig. 3 [file 44318_2026_788_MOESM5_ESM.zip › Figure 3/Figure 3H,3I/Figure 3H.tif]

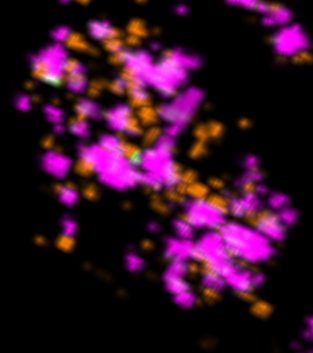

Supplement: Supplementary file 5 — Source data Fig. 3 [file 44318_2026_788_MOESM5_ESM.zip › Figure 3/Figure 3H,3I/Figure 3I.TIFF]

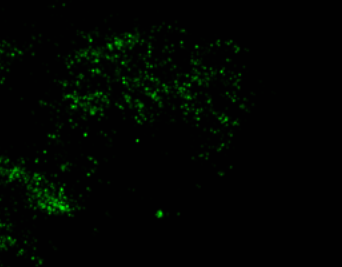

Supplement: Supplementary file 6 — Source data Fig. 4 [file 44318_2026_788_MOESM6_ESM.zip › Figure 4/4B,4D/Figure 4B.wdr5.KO.tif]

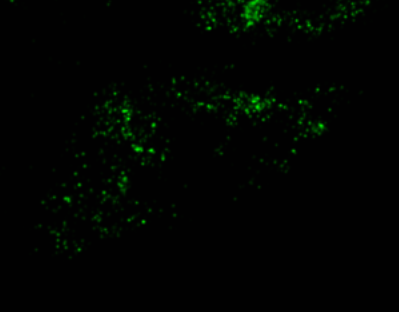

Supplement: Supplementary file 6 — Source data Fig. 4 [file 44318_2026_788_MOESM6_ESM.zip › Figure 4/4B,4D/Figure 4B.wdr5.NTC-cai.tif]

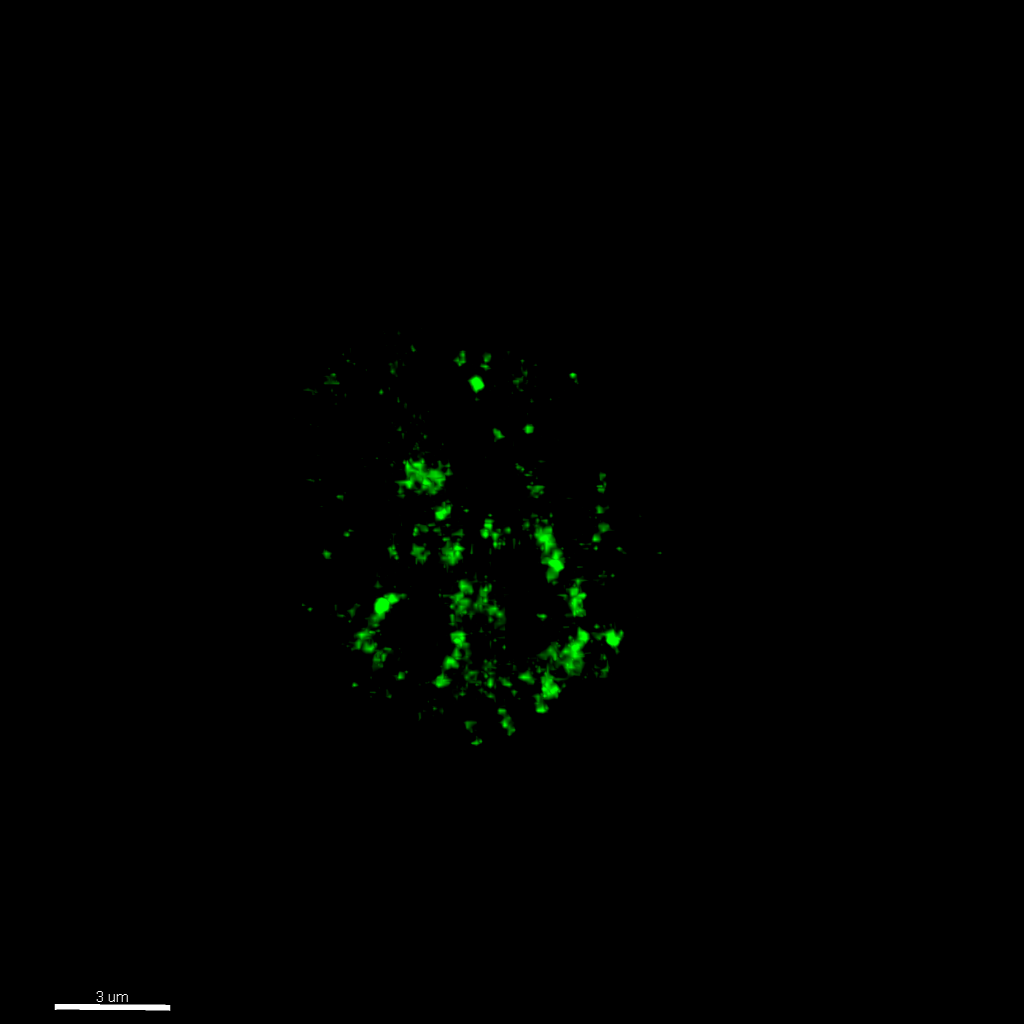

Supplement: Supplementary file 6 — Source data Fig. 4 [file 44318_2026_788_MOESM6_ESM.zip › Figure 4/4B,4D/Figure 4D.RING1B.KO.tif]

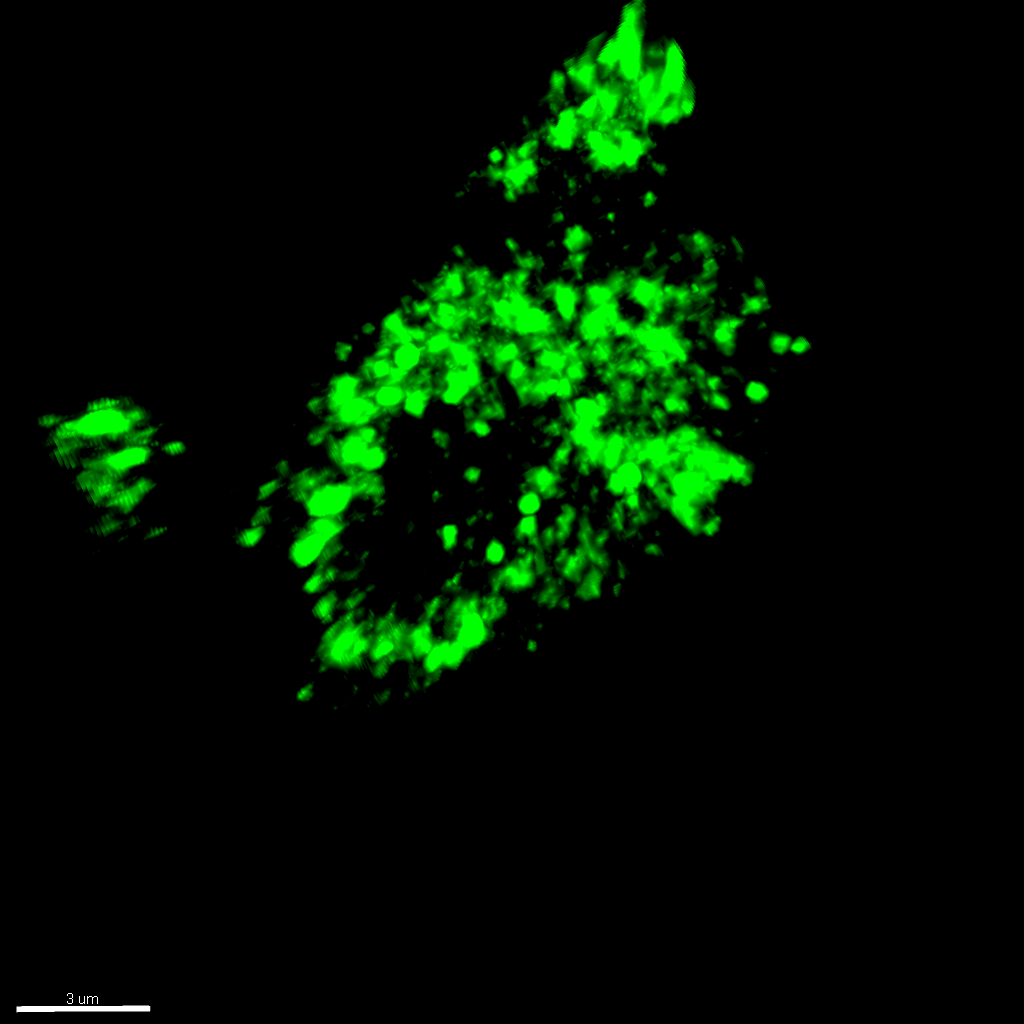

Supplement: Supplementary file 6 — Source data Fig. 4 [file 44318_2026_788_MOESM6_ESM.zip › Figure 4/4B,4D/Figure 4D.RING1B.NTC.tif]

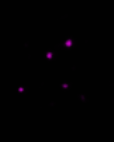

Supplement: Supplementary file 7 — Source data Fig. 5 [file 44318_2026_788_MOESM7_ESM.zip › Figure 5/Figure 5E/Rybp++.tif]

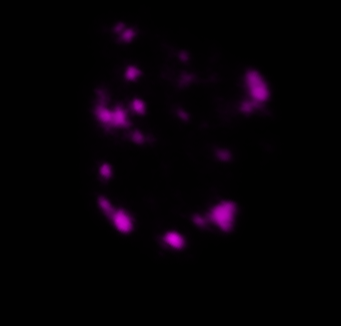

Supplement: Supplementary file 7 — Source data Fig. 5 [file 44318_2026_788_MOESM7_ESM.zip › Figure 5/Figure 5E/Rybp--.tif]

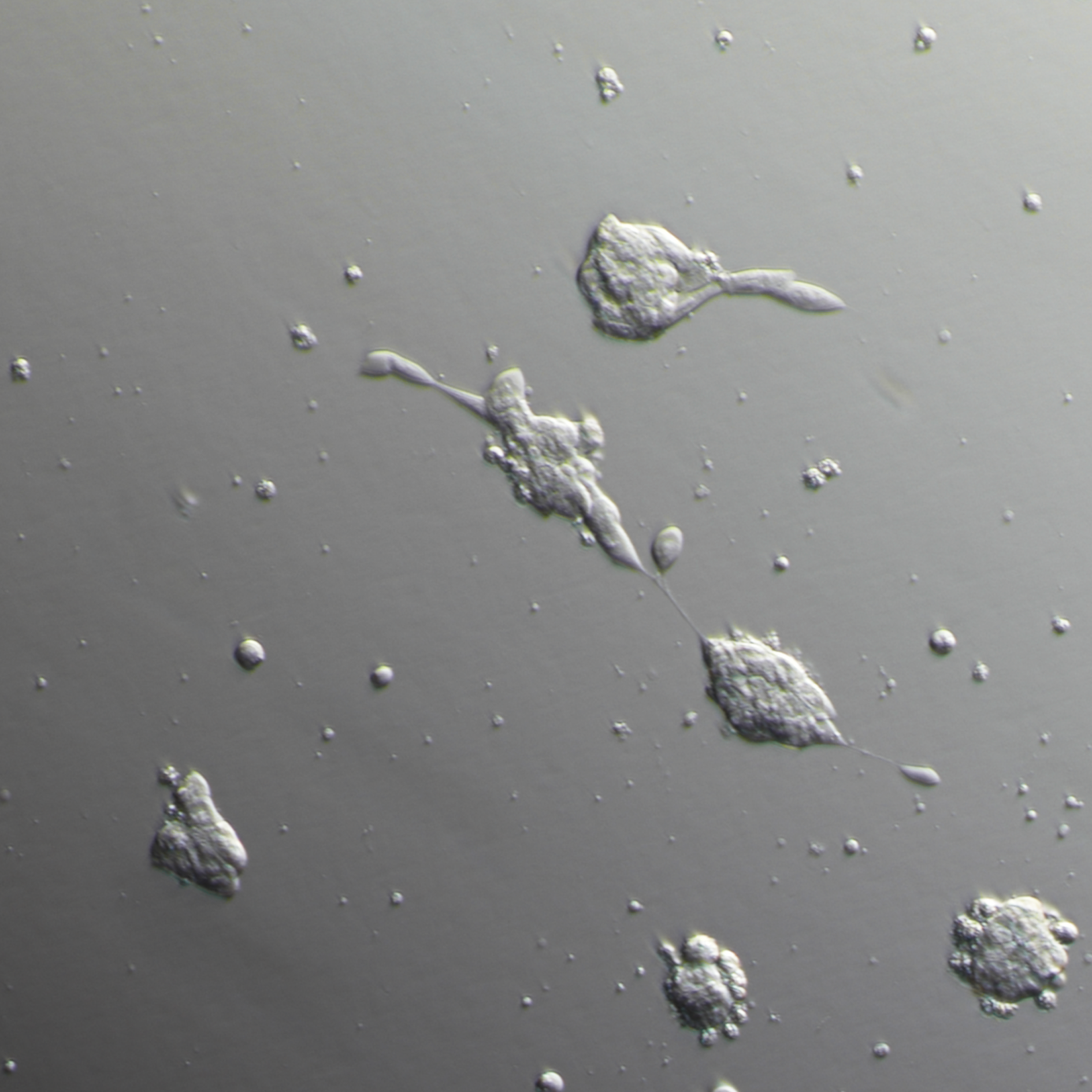

Supplement: Supplementary file 7 — Source data Fig. 5 [file 44318_2026_788_MOESM7_ESM.zip › Figure 5/Figure 5H/RYBPKO-D2.tif]

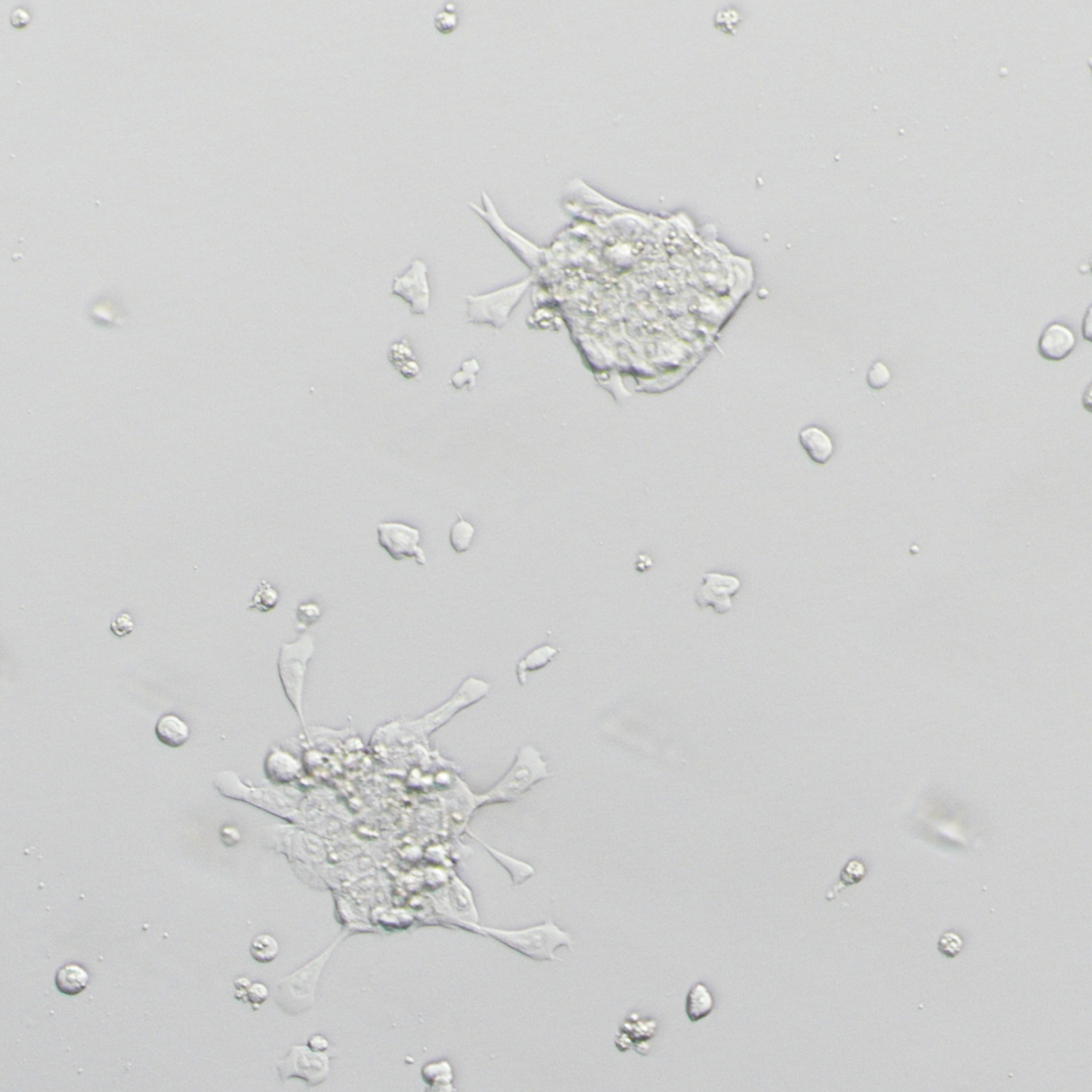

Supplement: Supplementary file 7 — Source data Fig. 5 [file 44318_2026_788_MOESM7_ESM.zip › Figure 5/Figure 5H/RYBPKO-D3.tif]

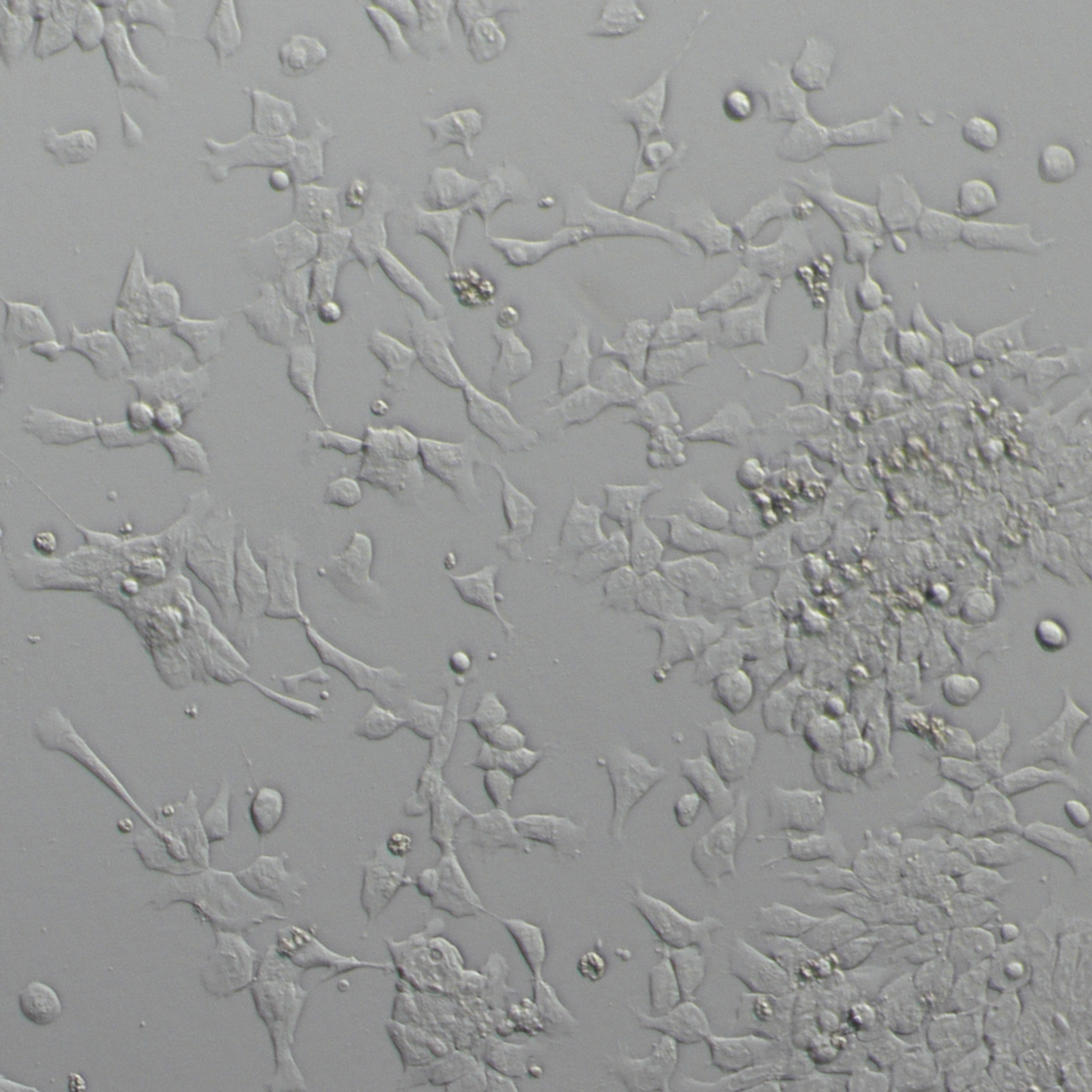

Supplement: Supplementary file 7 — Source data Fig. 5 [file 44318_2026_788_MOESM7_ESM.zip › Figure 5/Figure 5H/RYBPKO-D4.tif]

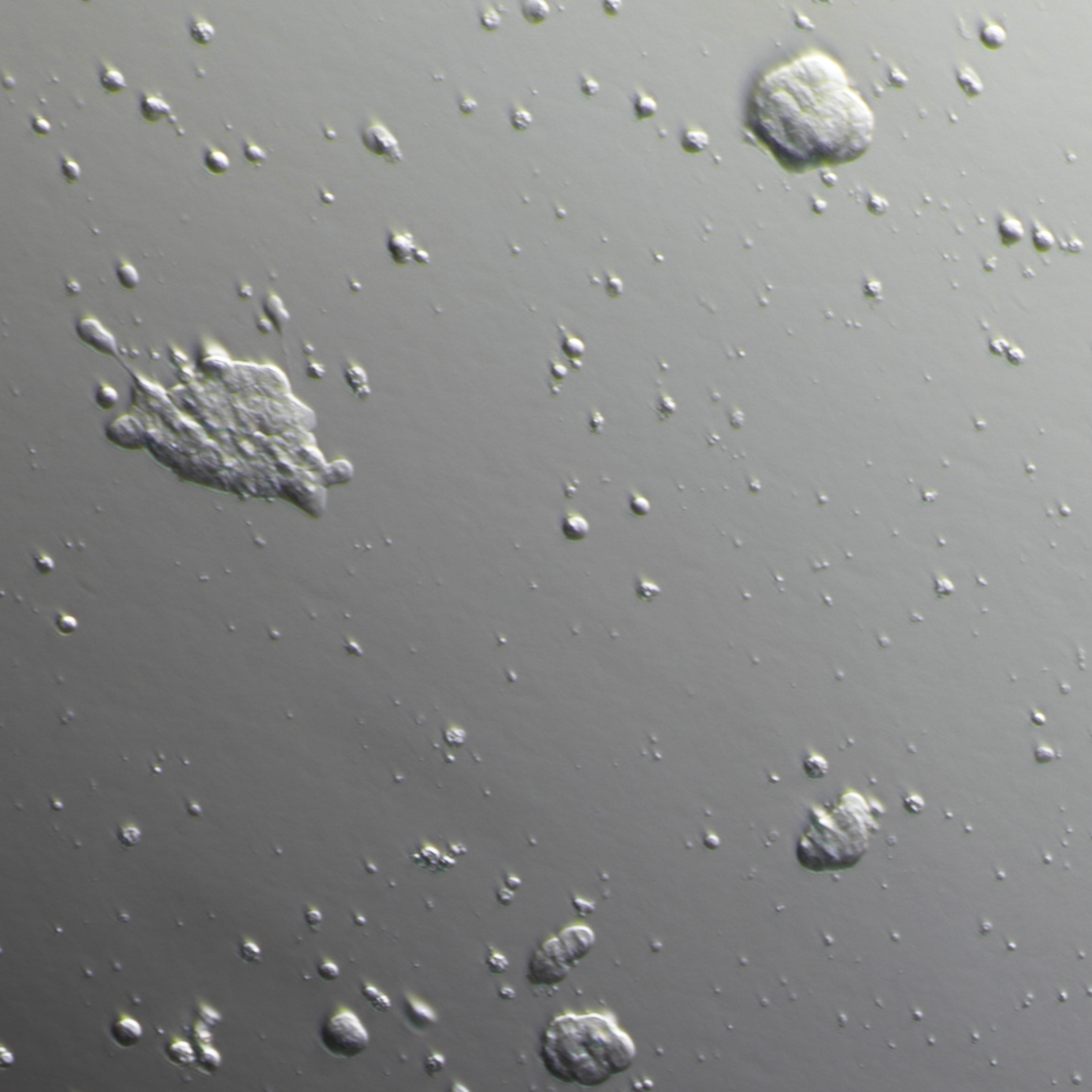

Supplement: Supplementary file 7 — Source data Fig. 5 [file 44318_2026_788_MOESM7_ESM.zip › Figure 5/Figure 5H/WT-D2.tif]

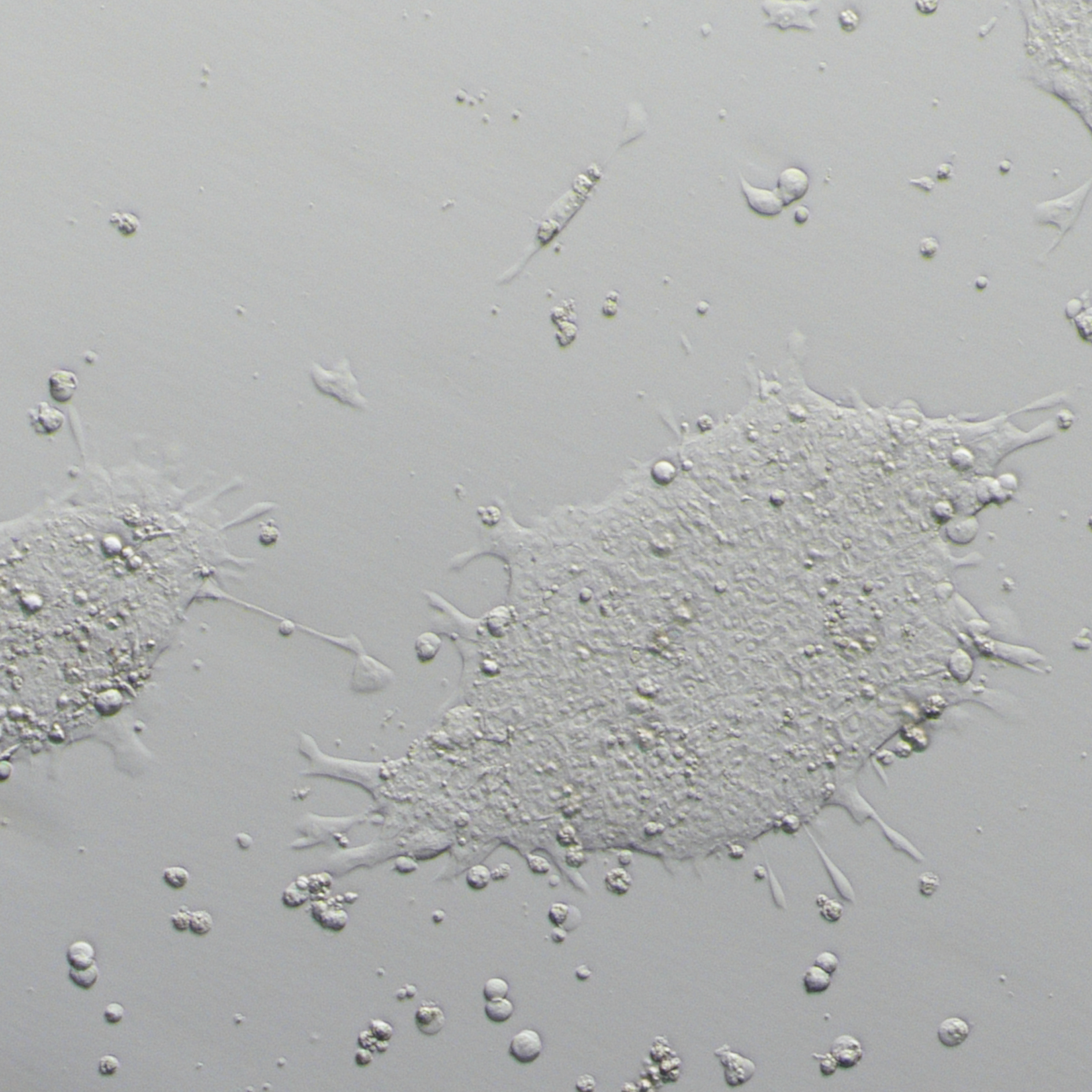

Supplement: Supplementary file 7 — Source data Fig. 5 [file 44318_2026_788_MOESM7_ESM.zip › Figure 5/Figure 5H/WT-D3.tif]

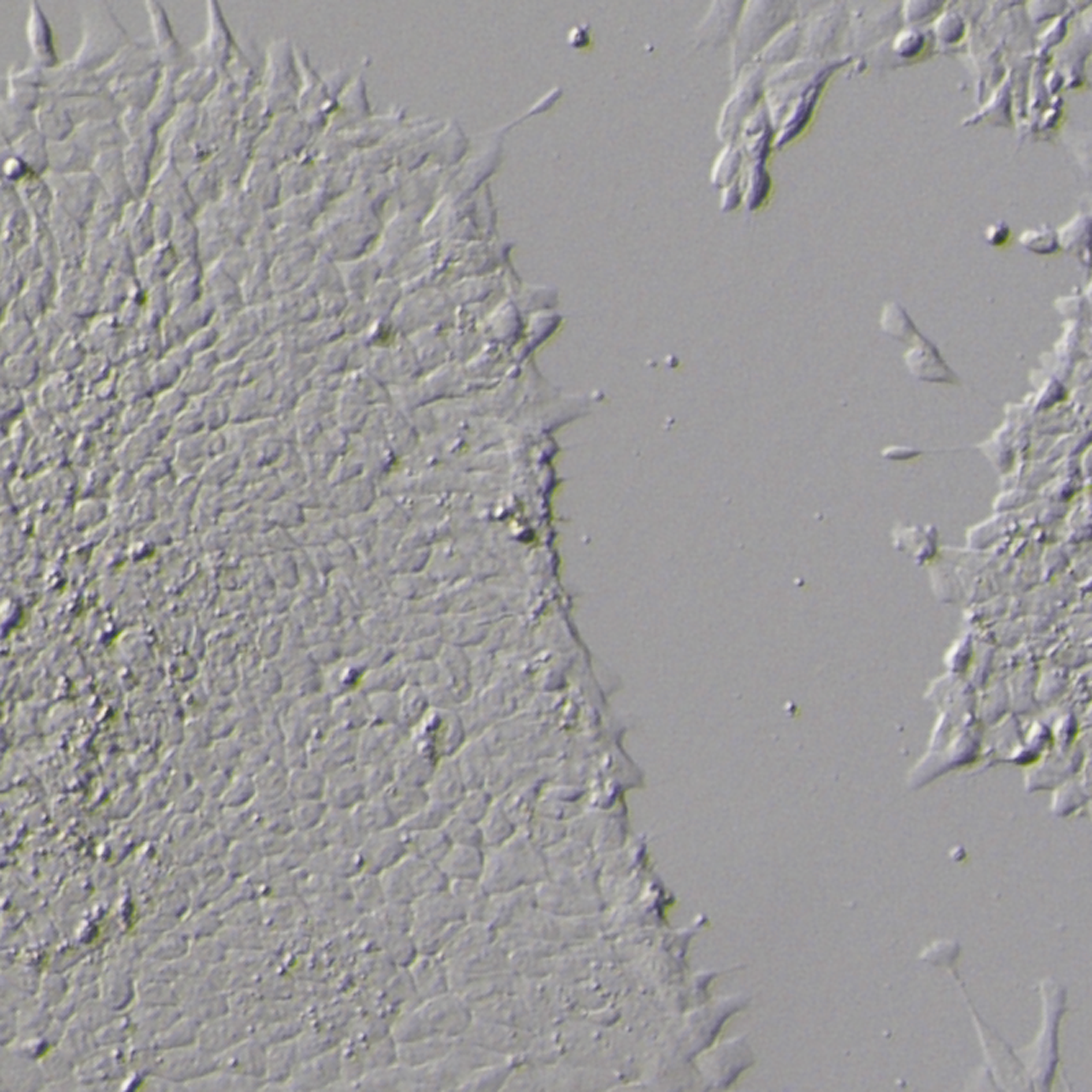

Supplement: Supplementary file 7 — Source data Fig. 5 [file 44318_2026_788_MOESM7_ESM.zip › Figure 5/Figure 5H/WT-D4.tif]

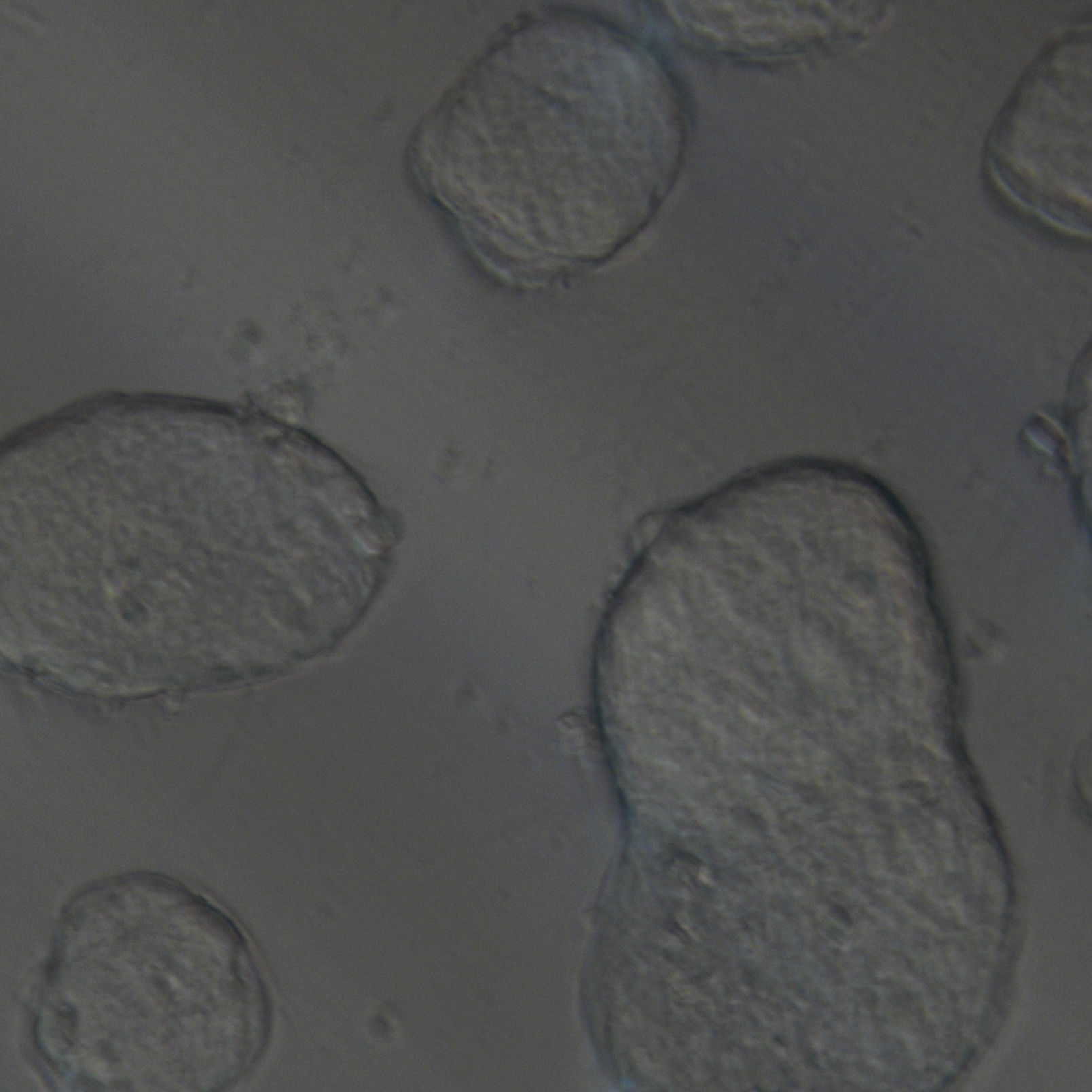

Supplement: Supplementary file 8 — Source data Fig. 6 [file 44318_2026_788_MOESM8_ESM.zip › Figure 6/Figure 6E/Rybp++.bright.tif]

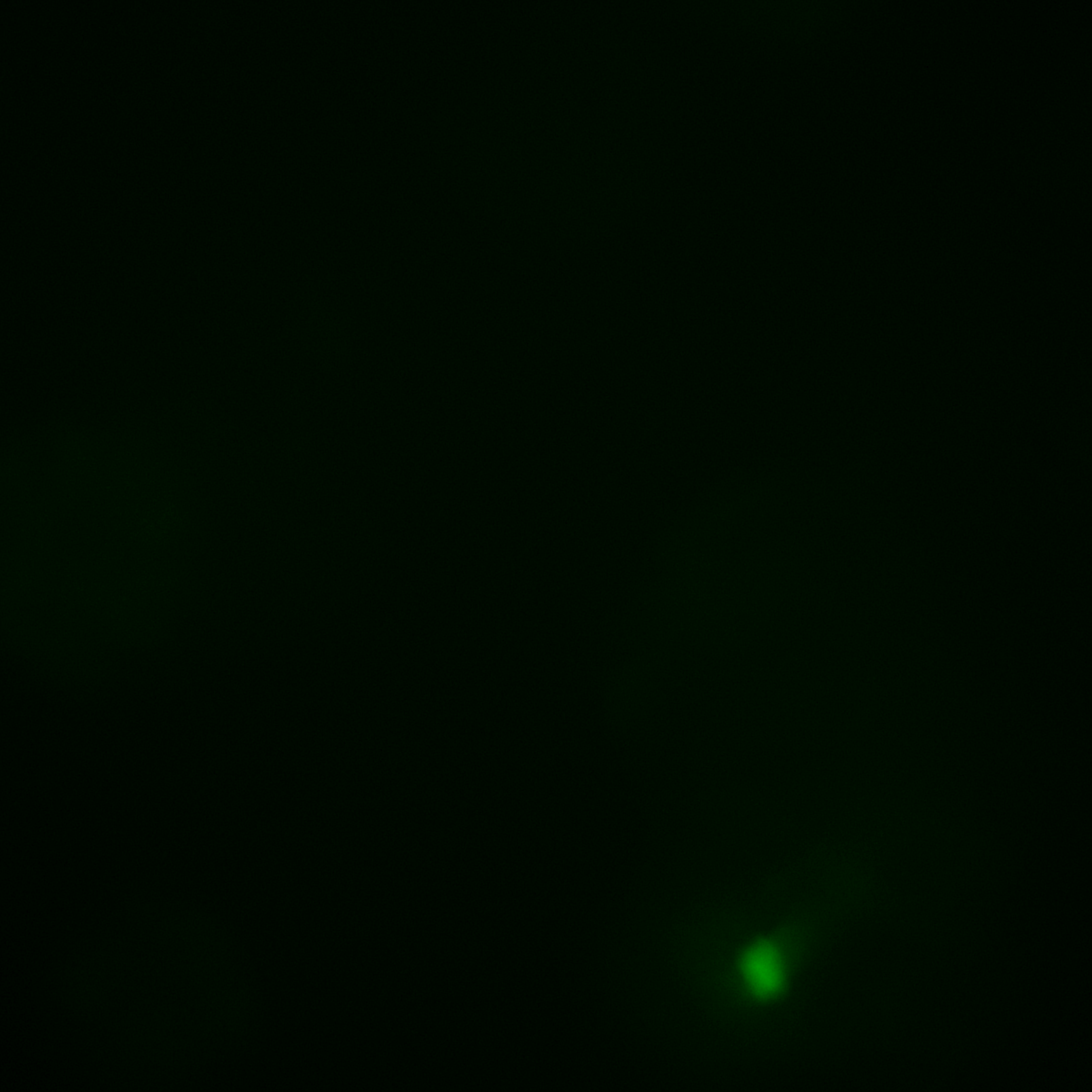

Supplement: Supplementary file 8 — Source data Fig. 6 [file 44318_2026_788_MOESM8_ESM.zip › Figure 6/Figure 6E/Rybp++.GFP.tif]

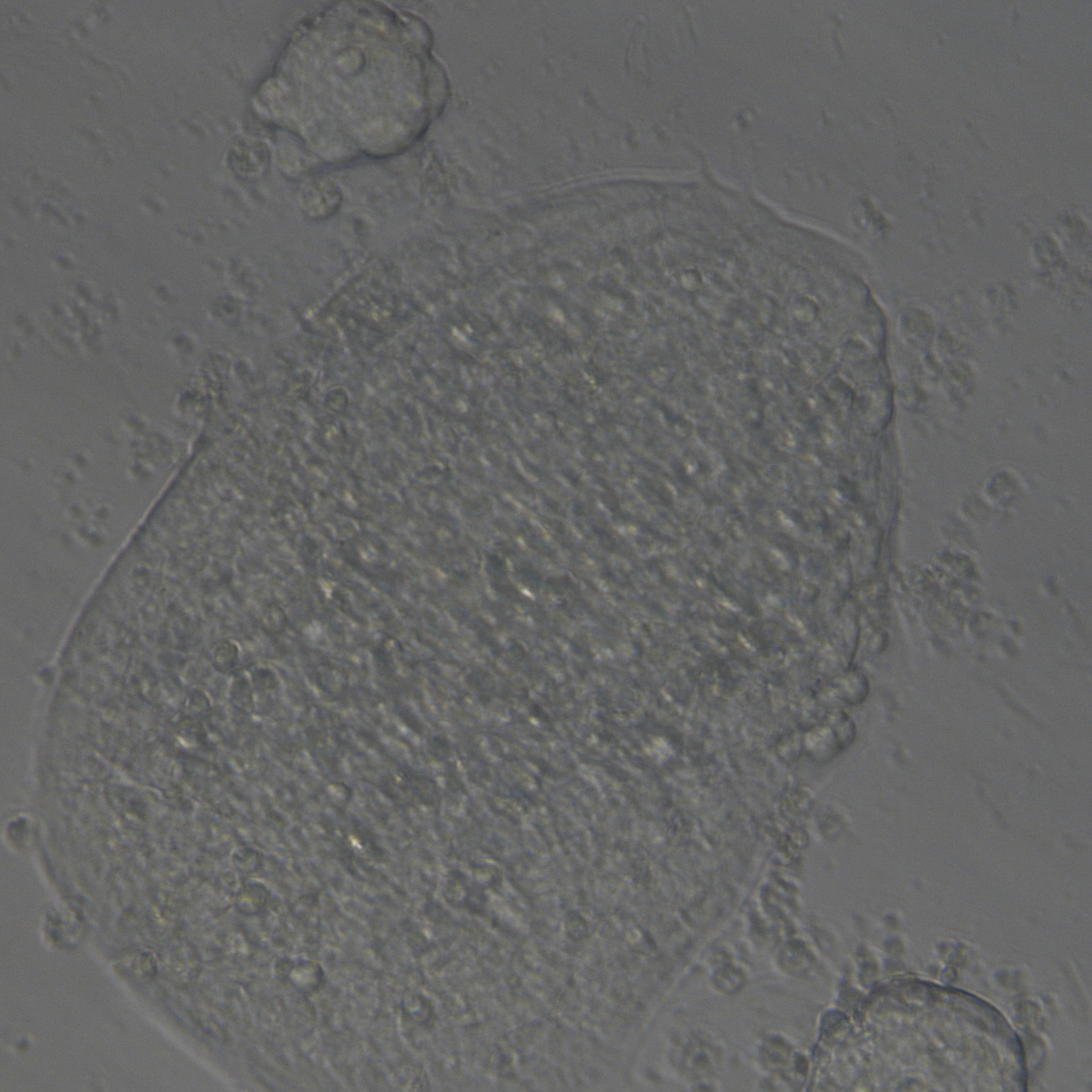

Supplement: Supplementary file 8 — Source data Fig. 6 [file 44318_2026_788_MOESM8_ESM.zip › Figure 6/Figure 6E/Rybp--.bright.tif]

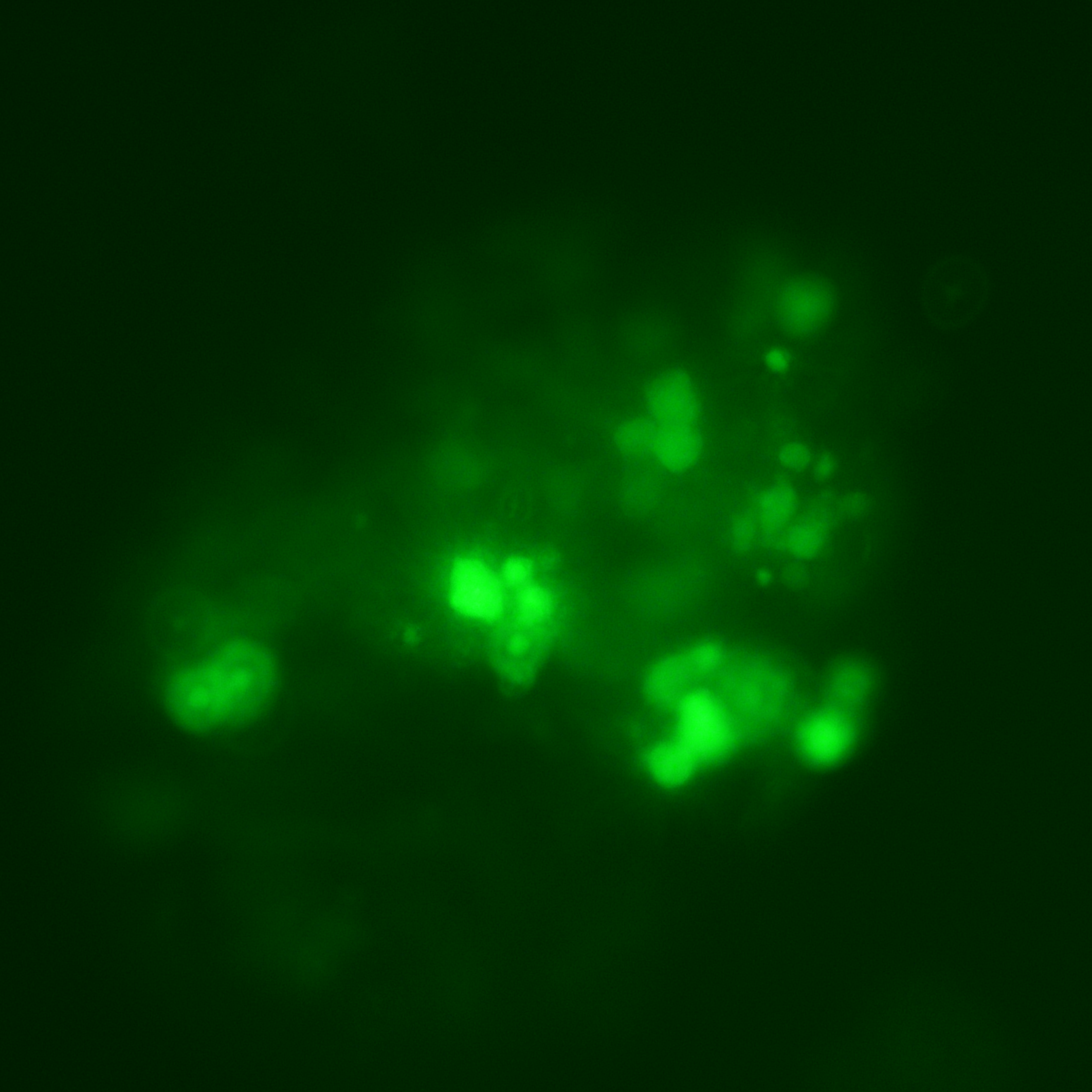

Supplement: Supplementary file 8 — Source data Fig. 6 [file 44318_2026_788_MOESM8_ESM.zip › Figure 6/Figure 6E/Rybp--.GFP.tif]

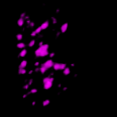

Supplement: Supplementary file 8 — Source data Fig. 6 [file 44318_2026_788_MOESM8_ESM.zip › Figure 6/Figure 6L/shBrca1.tif]

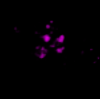

Supplement: Supplementary file 8 — Source data Fig. 6 [file 44318_2026_788_MOESM8_ESM.zip › Figure 6/Figure 6L/shEV.tif]

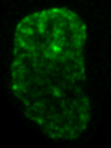

Supplement: Supplementary file 9 — Figure EV1 Source Data [file 44318_2026_788_MOESM9_ESM.zip › Figure EV1/Figure EV1D/RYBP-EGFP.bleach 0s.tif]

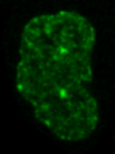

Supplement: Supplementary file 9 — Figure EV1 Source Data [file 44318_2026_788_MOESM9_ESM.zip › Figure EV1/Figure EV1D/RYBP-EGFP.bleach 20s.tif]

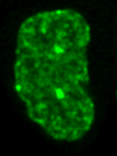

Supplement: Supplementary file 9 — Figure EV1 Source Data [file 44318_2026_788_MOESM9_ESM.zip › Figure EV1/Figure EV1D/RYBP-EGFP.pre-bleach.tif]

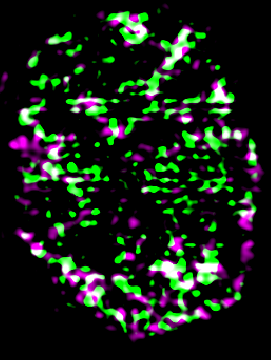

Supplement: Supplementary file 9 — Figure EV1 Source Data [file 44318_2026_788_MOESM9_ESM.zip › Figure EV1/Figure EV1E/merge.RBBP5.tif]

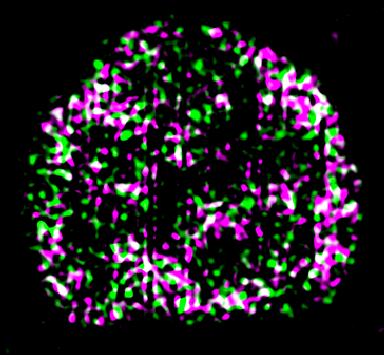

Supplement: Supplementary file 9 — Figure EV1 Source Data [file 44318_2026_788_MOESM9_ESM.zip › Figure EV1/Figure EV1E/merge.Ash2l.tif]

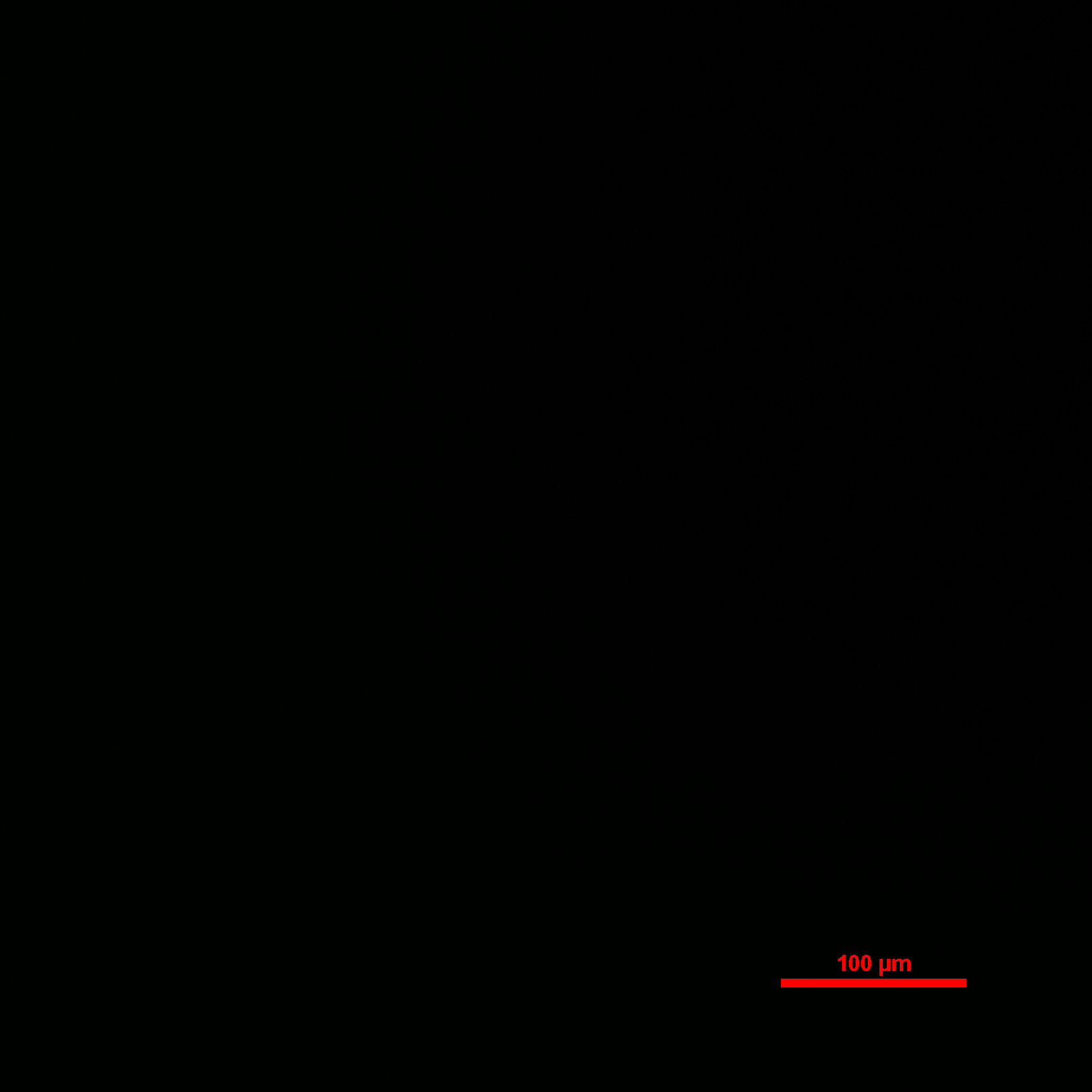

Supplement: Supplementary file 11 — Figure EV3 Source Data [file 44318_2026_788_MOESM11_ESM.zip › Figure EV3/Figure EV3B/DOX-.EGFP.tif]

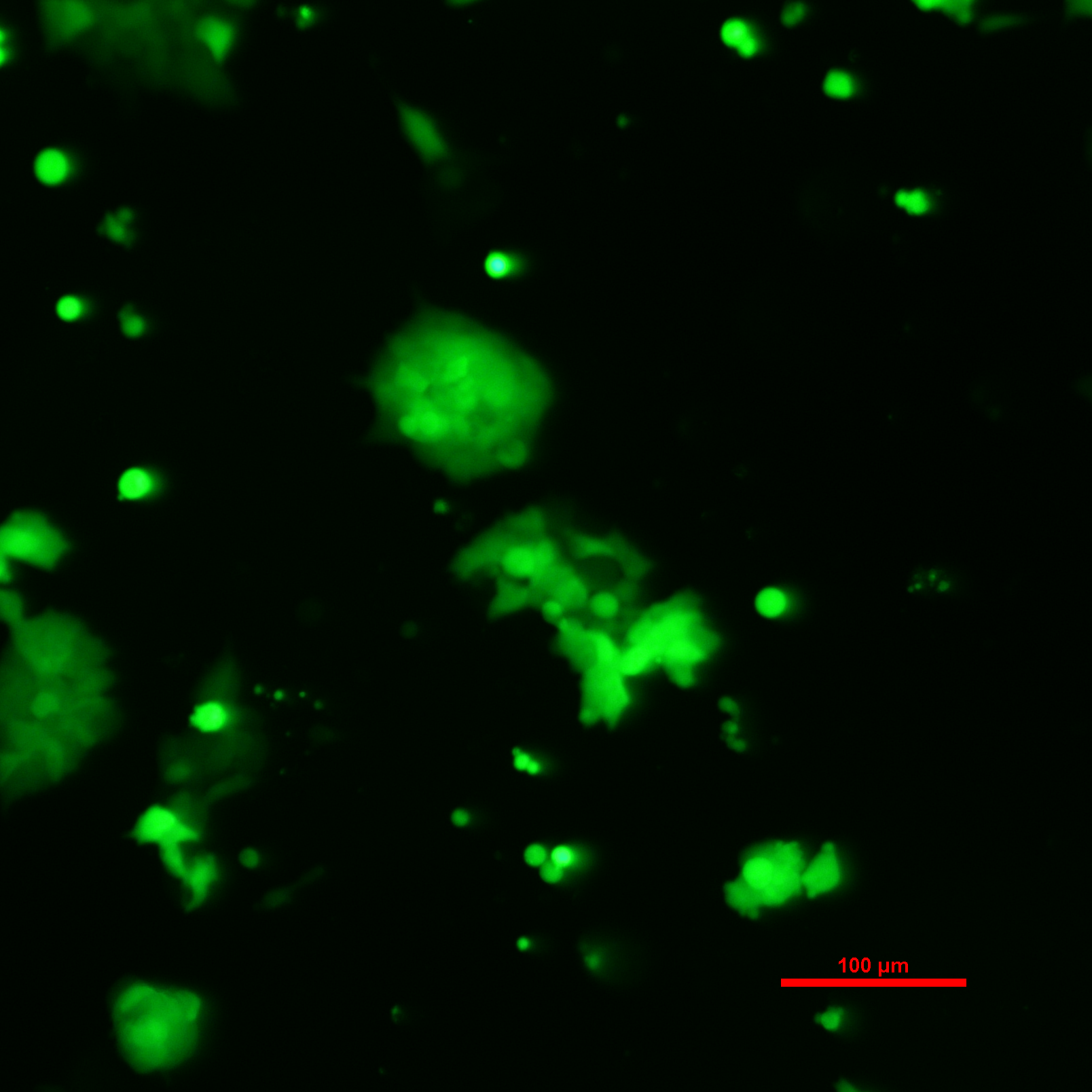

Supplement: Supplementary file 11 — Figure EV3 Source Data [file 44318_2026_788_MOESM11_ESM.zip › Figure EV3/Figure EV3B/DOX+.EGFP.tif]

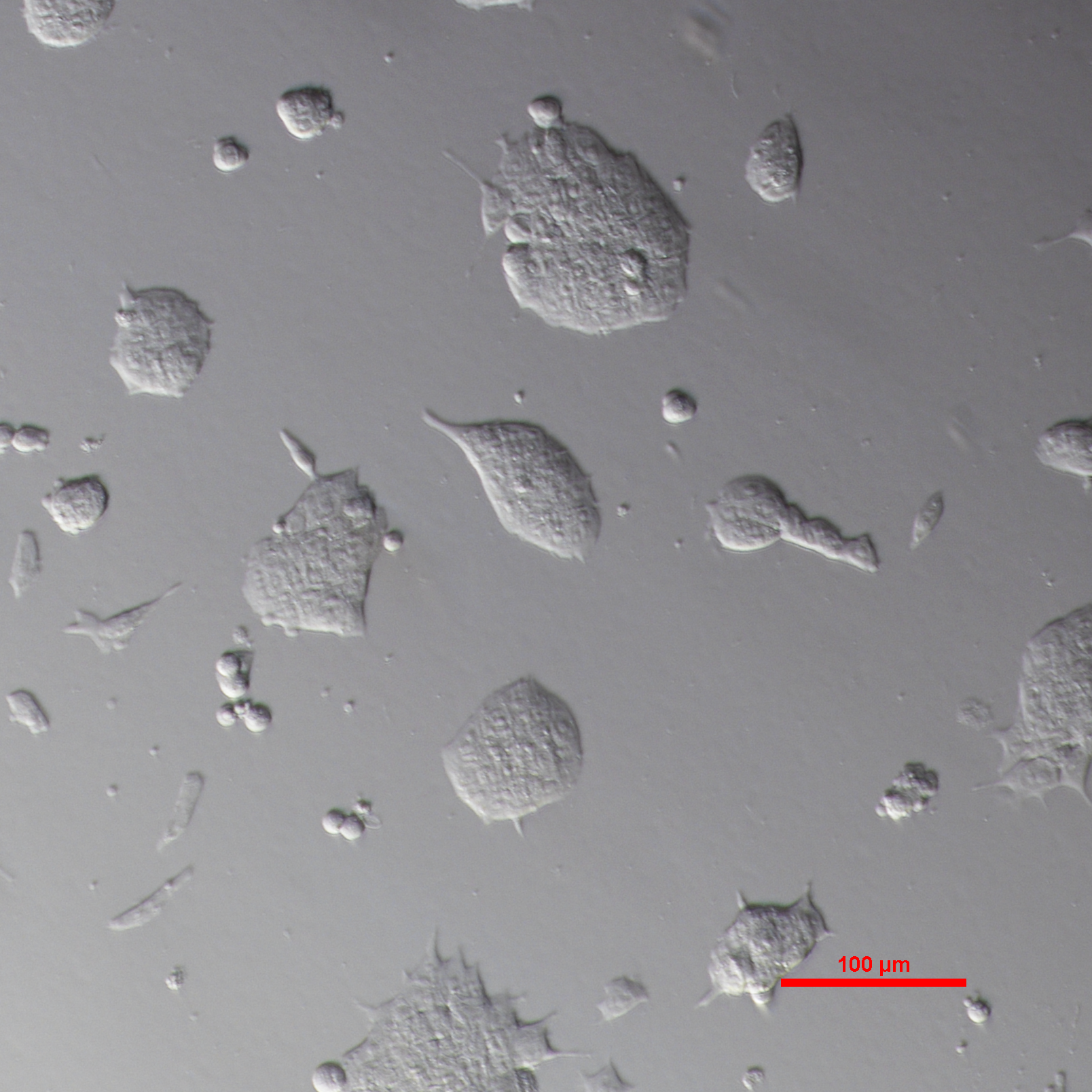

Supplement: Supplementary file 11 — Figure EV3 Source Data [file 44318_2026_788_MOESM11_ESM.zip › Figure EV3/Figure EV3B/DOX-.bright.tif]

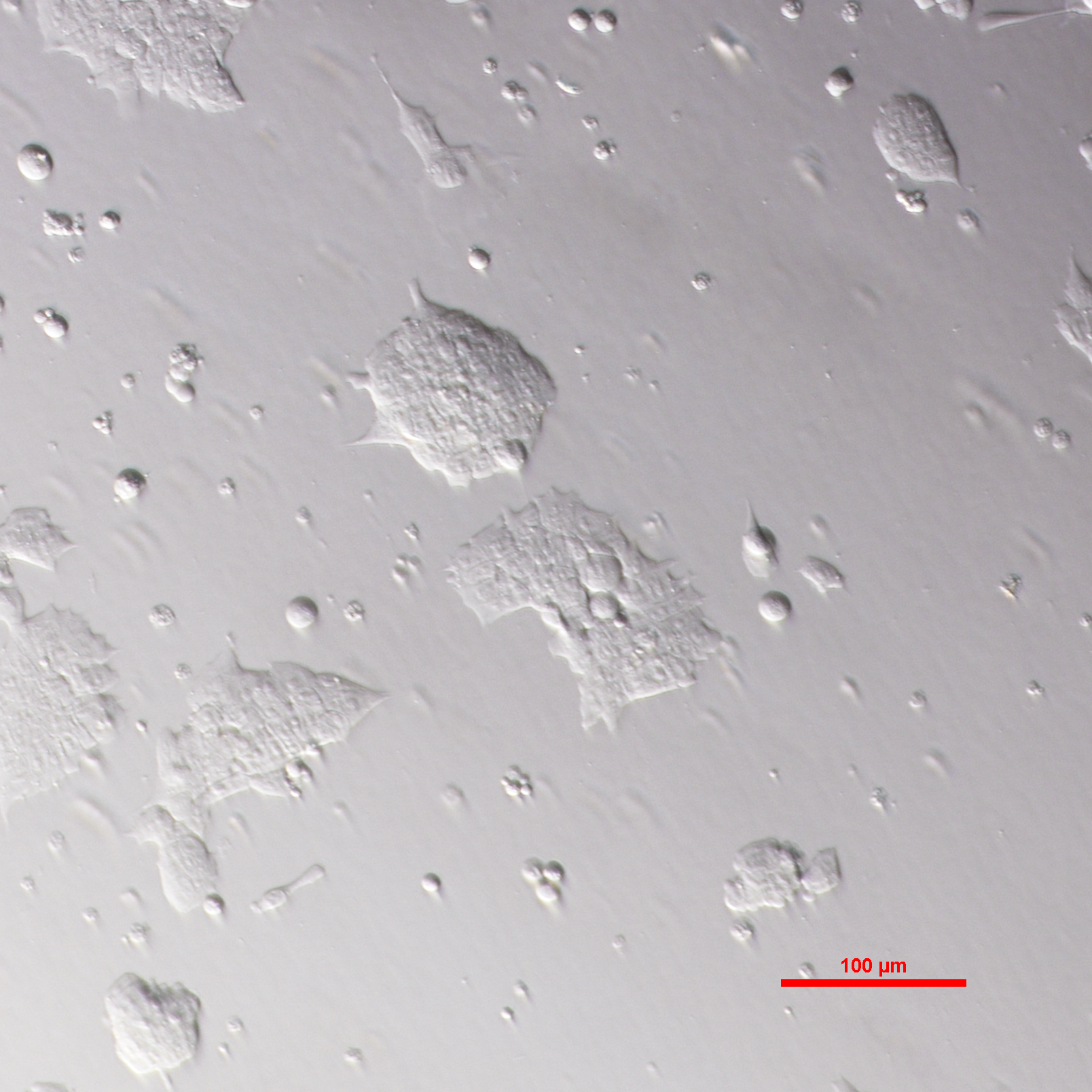

Supplement: Supplementary file 11 — Figure EV3 Source Data [file 44318_2026_788_MOESM11_ESM.zip › Figure EV3/Figure EV3B/DOX+.bright.tif]
