# Supplementary material for: RYBP regulates selective genomic binding of TrxG and PcG components in embryonic stem cell fate control
Source: EMBO J. 2026 Apr 28;45(11):3808–32. doi: 10.1038/s44318-026-00788-y (PMC13226663; doi:10.1038/s44318-026-00788-y)
Supplement: Supplementary file 9 — Figure EV1 Source Data [file 44318_2026_788_MOESM9_ESM.zip › Figure EV1/Figure EV1B-1C.pptx]

## Slide 1
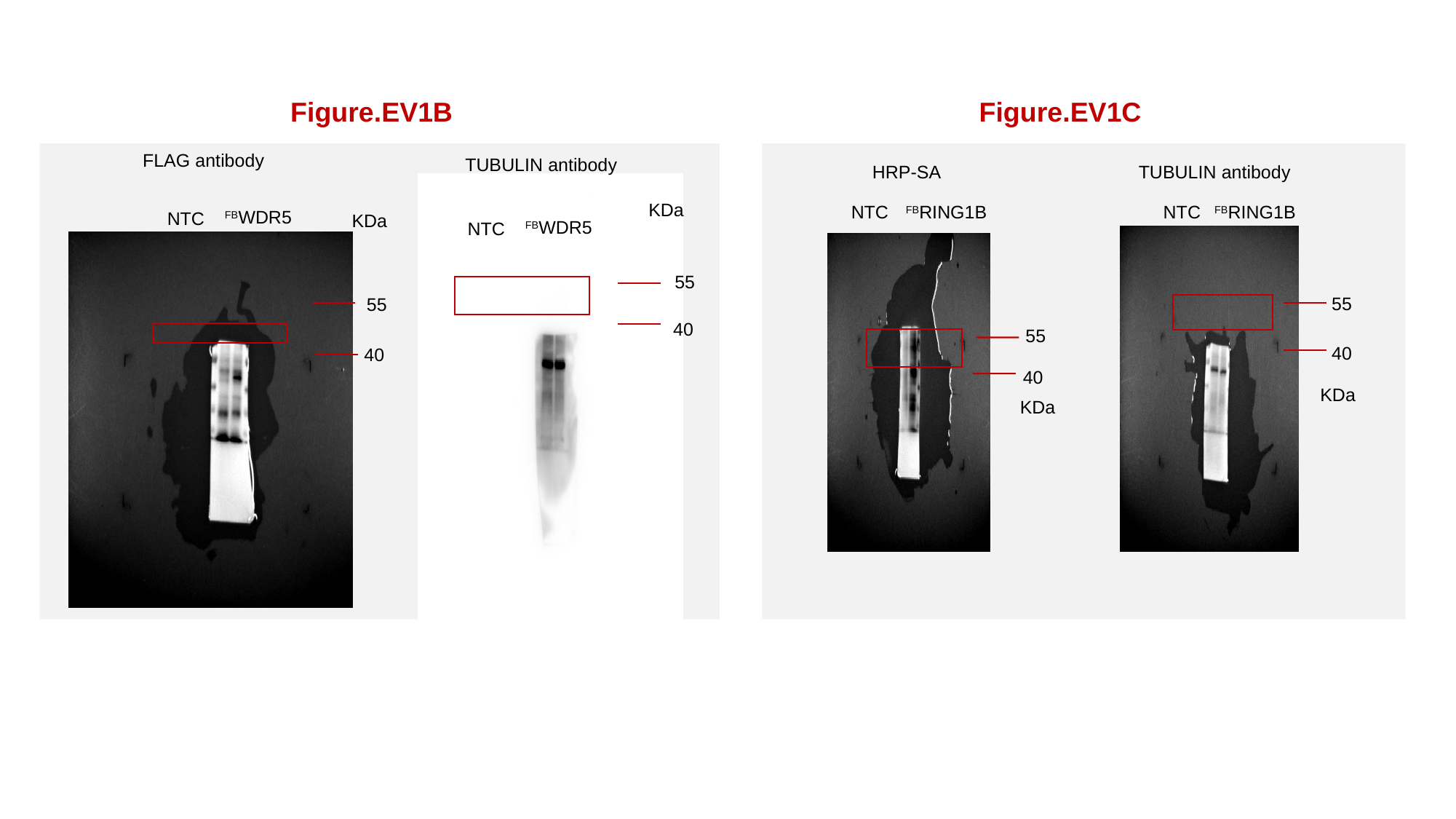

Figure.EV1B
Figure.EV1C
FLAG antibody
TUBULIN antibody
KDa
FBWDR5
NTC
KDa
FBWDR5
NTC
55
55
40
40
HRP-SA
TUBULIN antibody
NTC
FBRING1B
NTC
FBRING1B
55
40
55
40
KDa
KDa
