# Supplementary material for: RYBP regulates selective genomic binding of TrxG and PcG components in embryonic stem cell fate control
Source: EMBO J. 2026 Apr 28;45(11):3808–32. doi: 10.1038/s44318-026-00788-y (PMC13226663; doi:10.1038/s44318-026-00788-y)
Supplement: Supplementary file 10 — Figure EV2 Source Data [file 44318_2026_788_MOESM10_ESM.zip › Figure EV2/Figure EV2A,2B.pptx]

## Slide 1
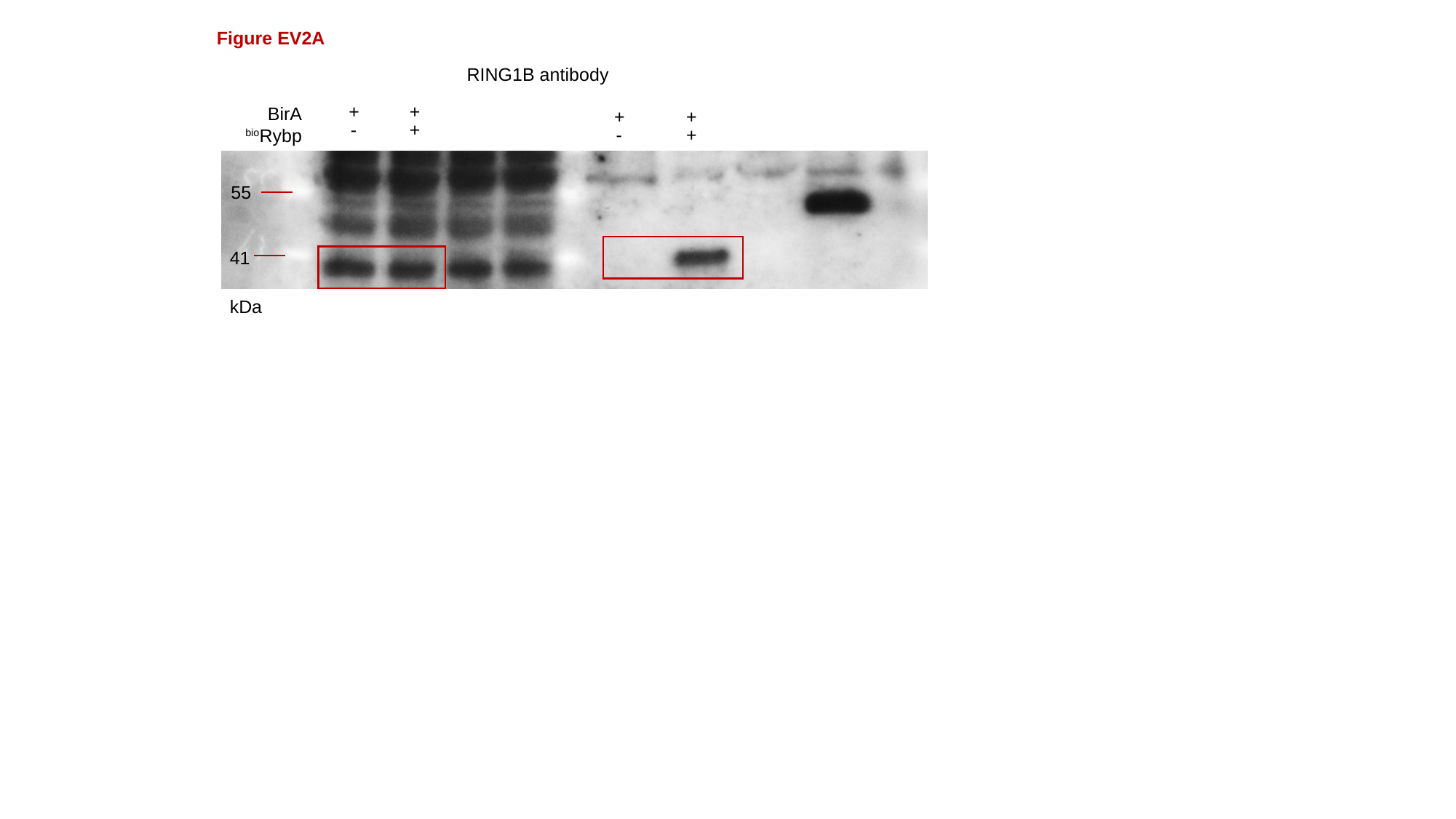

Figure EV2A
RING1B antibody
+
+
BirA
bioRybp
+
+
-
+
-
+
55
41
kDa

## Slide 2
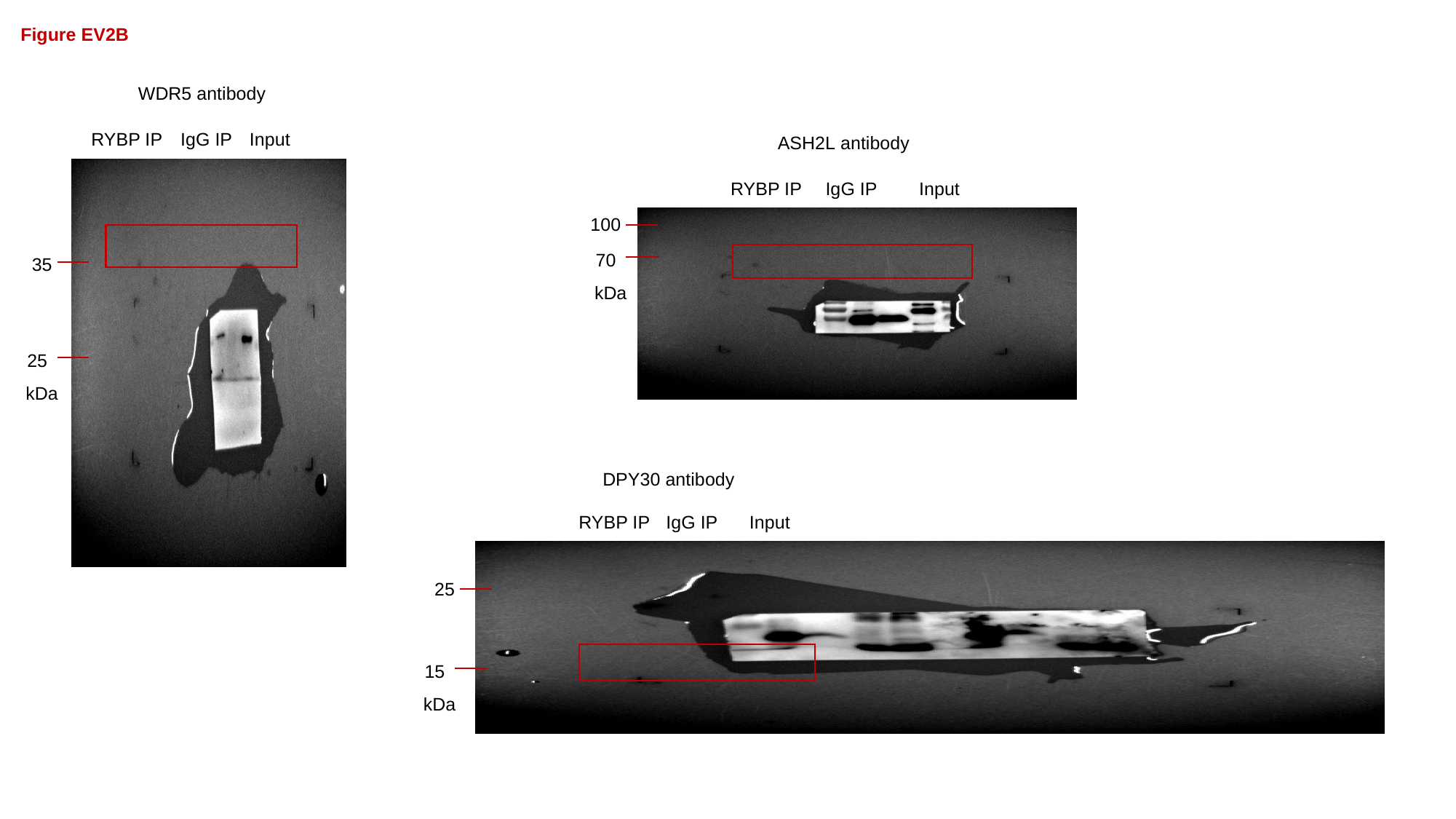

Figure EV2B
WDR5 antibody
RYBP IP
IgG IP
Input
ASH2L antibody
RYBP IP
IgG IP
Input
100
70
35
kDa
25
kDa
DPY30 antibody
RYBP IP
IgG IP
Input
25
15
kDa
