# Supplementary material for: RYBP regulates selective genomic binding of TrxG and PcG components in embryonic stem cell fate control
Source: EMBO J. 2026 Apr 28;45(11):3808–32. doi: 10.1038/s44318-026-00788-y (PMC13226663; doi:10.1038/s44318-026-00788-y)
Supplement: Supplementary file 12 — Expanded View Figures [file 44318_2026_788_MOESM12_ESM.pdf]

Expanded View Figures

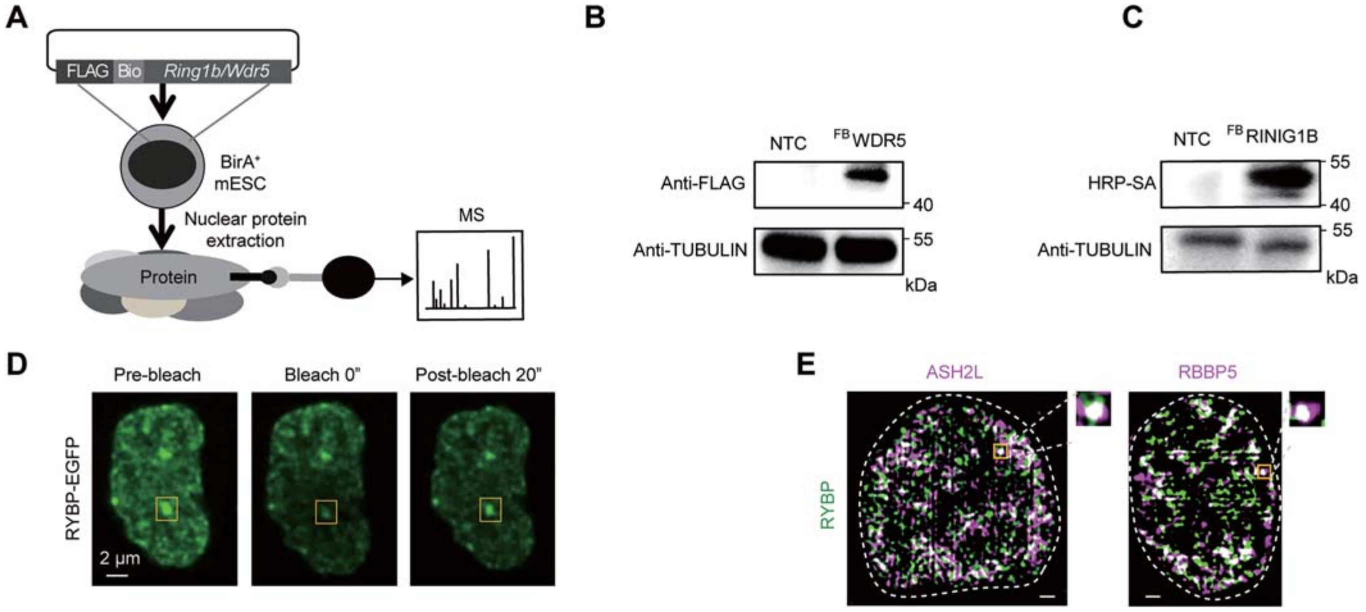

Figure EV1. RYBP co-localizes with TrxG and PcG components in condensates.

(**A**) IP-MS schematic diagram for identifying WDR5 and RING1B protein Interactome. (**B**, **C**) Western blot showing the exogenous expression of FLAG-biotin-tagged WDR5 (^{FB}WDR5) (**B**) and FLAG-biotin-tagged RING1B (^{FB}RING1B) (**C**). (**D**) Representative images of fluorescence recovery after photobleaching (FRAP) in mESCs expressing exogenous RYBP-EGFP. (**E**) Representative immunofluorescence images showing the co-localization between RYBP and TrxG components (ASH2L, RBBP5). Scale bar denotes 2 μm. Source data are available online for this figure.

 © The Author(s)

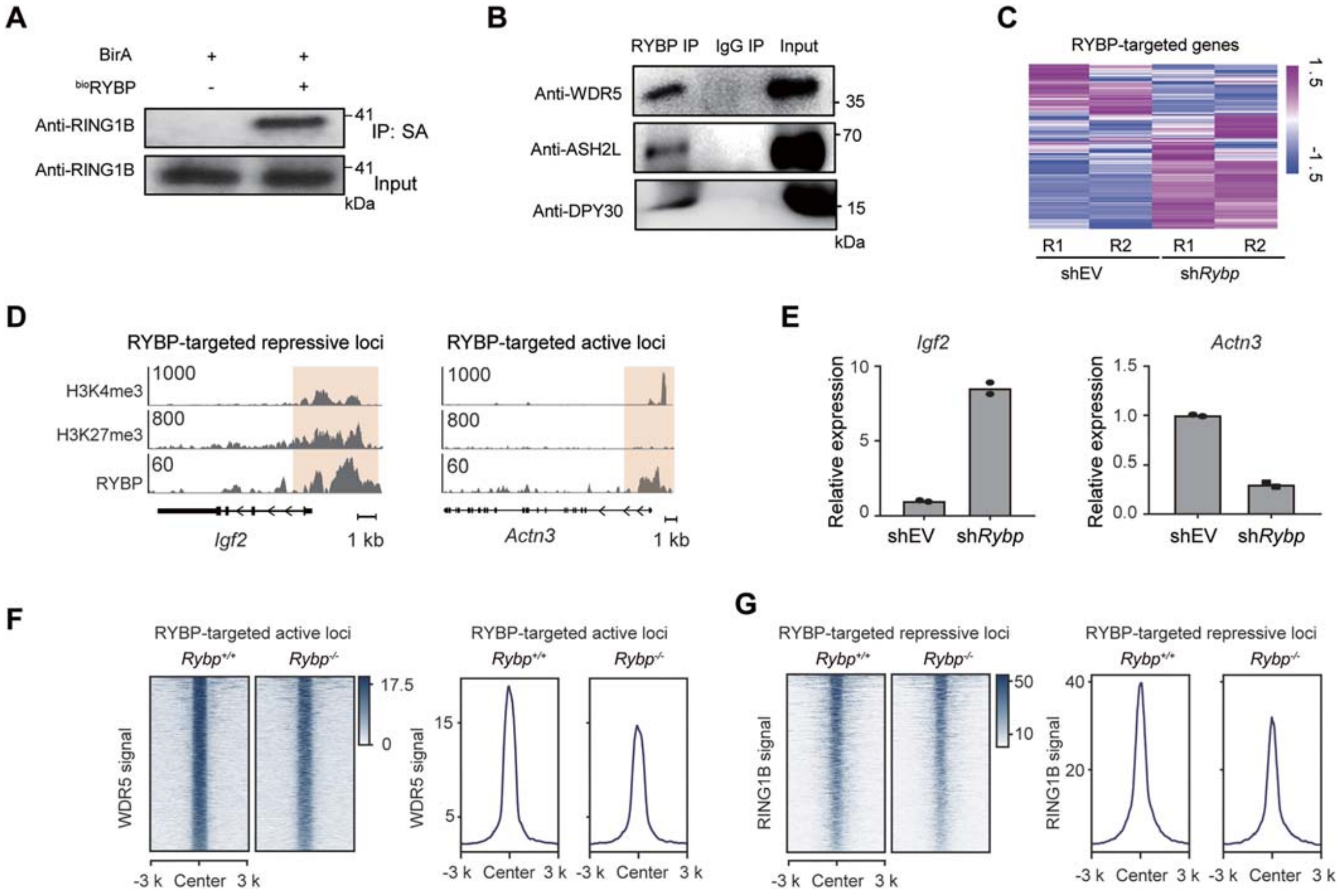

Figure EV2. RYBP is involved in both transcriptionally active and repressive functions.

(**A**, **B**) Co-IP validates the interaction between RYBP and RING1B (**A**), between RYBP and TrxG components (**B**) in ESCs. (**C**) Relative expression of RYBP-targeted genes in RYBP-deficiency ESCs (sh*Rybp*) compared with empty vector lentivirus-infected ESCs (sh*EV*). (**D**) Deposition of RYBP, H3K4me3 and H3K27me3 at the *Igf2* and *Actn3* locus. (**E**) The expression changes of *Igf2* and *Actn3* genes after *Rybp* knockdown, $n = 2$. (**F**, **G**) Heatmaps showing the ChIP signal of WDR5 at RYBP-targeted active loci (**F**), and RING1B at RYBP-targeted repressive loci after RYBP knockout (**G**). Source data are available online for this figure.

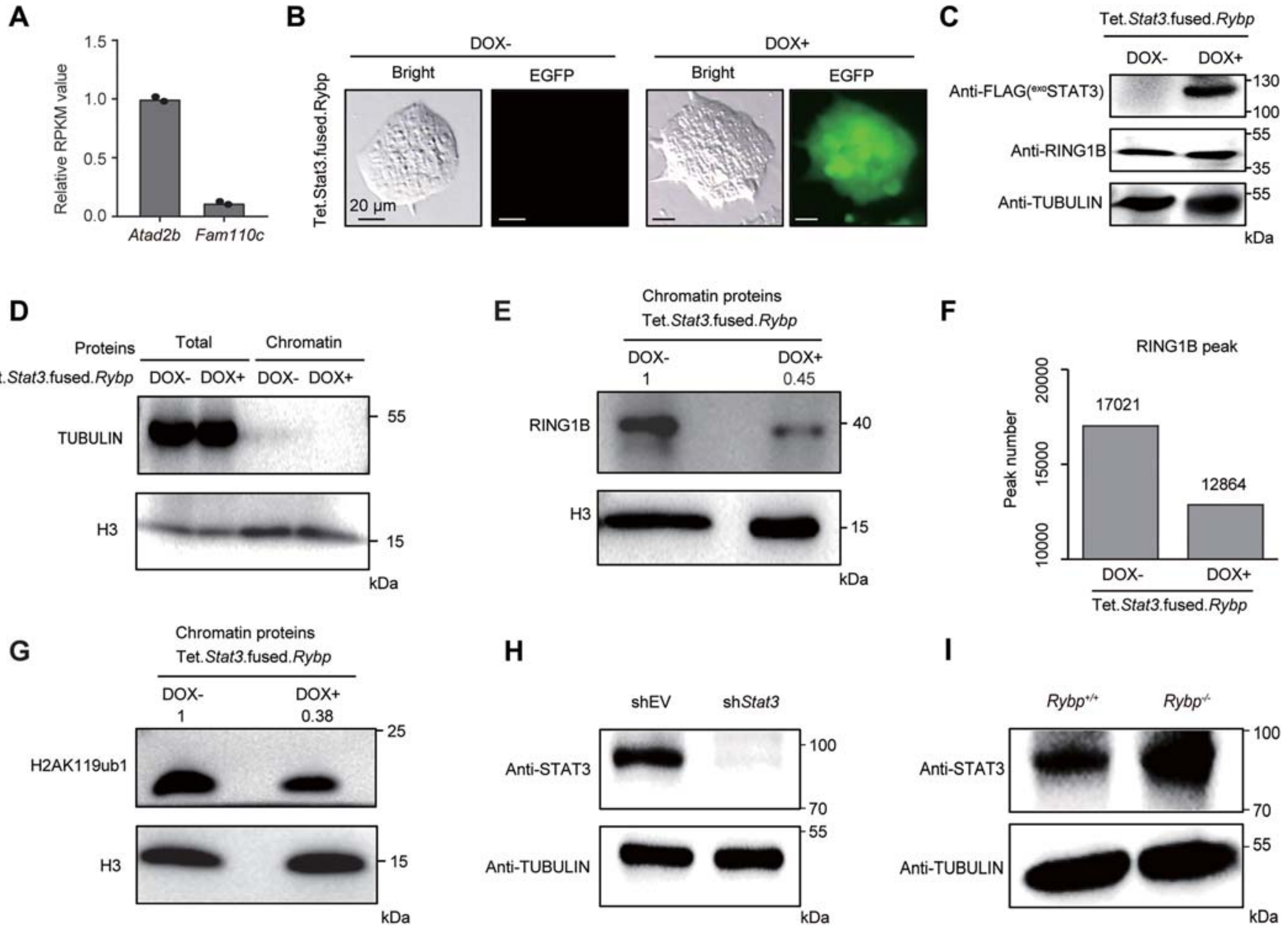

Figure EV3. STAT3 excludes RING1B on chromatin.

(**A**) The histogram showing the relative RPKM value of *Atad2b* and *Fam110c*, $n = 2$. (**B**, **C**) Representative fluorescence images and western blot verifying the successful establishment of Tet.*Stat3*.fused.*Rybp* cell line after DOX addition. (**D**) Western blot showing the level of TUBULIN and H3 from total and chromatin proteins. (**E**) Western blot showing the deposition of RING1B at chromatin after DOX treatment in Tet.*Stat3*.fused.*Rybp* cells. (**F**) Number of RING1B peaks before and after inducing expression of STAT3-fused RYBP protein. (**G**) Western blot showing the deposition of H2AK119ub1 at chromatin after DOX treatment in Tet.*Stat3*.fused.*Rybp* cells. (**H**, **I**) Western blot showing the expression of STAT3 after STAT3 knockdown (**H**) or RYBP depletion (**I**). Source data are available online for this figure.

 © The Author(s)

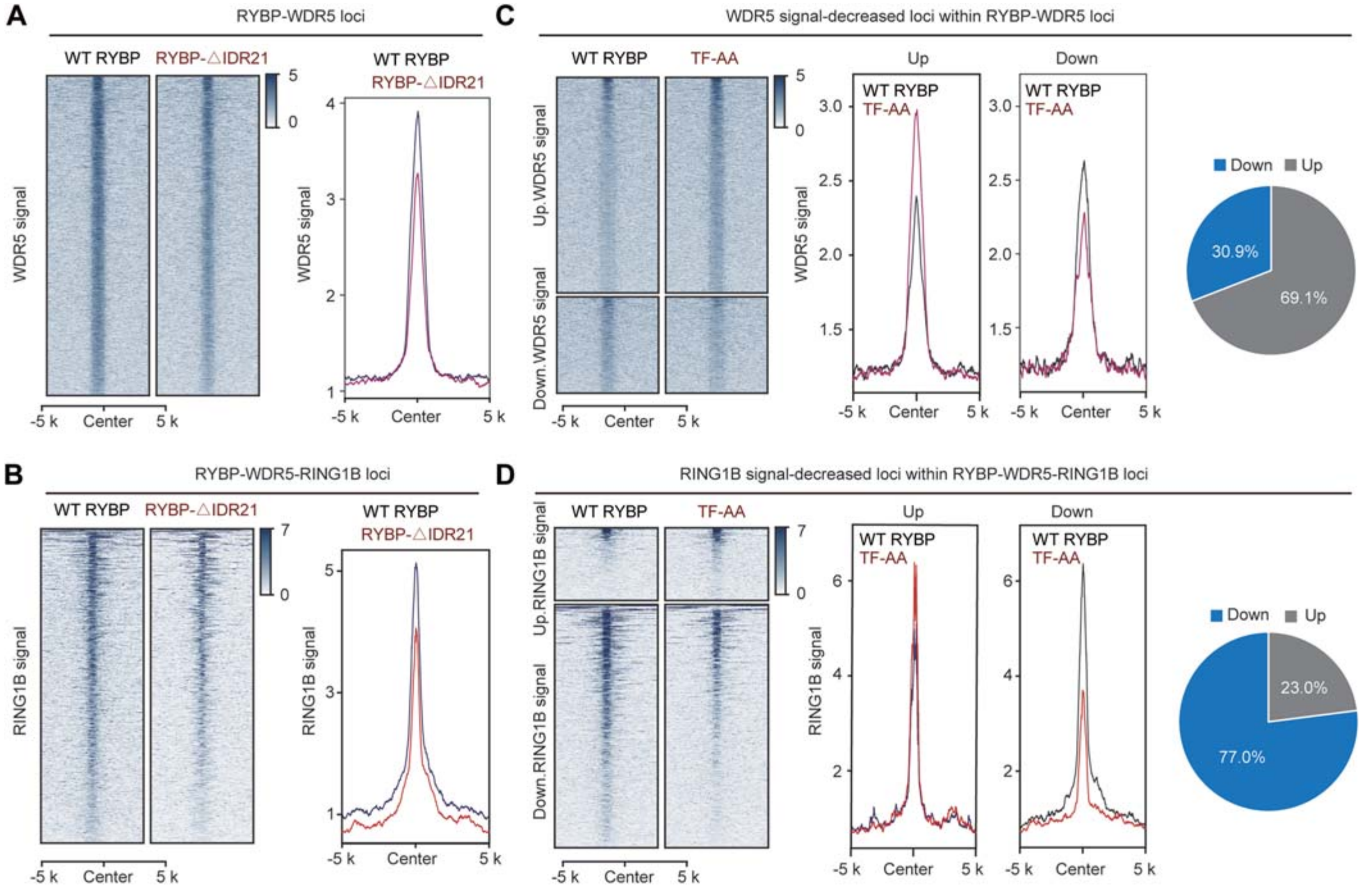

Figure EV4. RYBP depletion reduces the genomic deposition of H3K27ac and H3K27me3.

(**A**, **B**) The heatmap and curve graph showing the ChIP signal of WDR5 (**A**) and RING1B (**B**) at numerous loci following the phase disruption of RYBP. (**C**, **D**) Among the signal-decreased loci following RYBP phase disruption, the ratio of upregulation and downregulation of WDR5 (**C**) and RING1B (**D**) signals following the TF-AA mutation.
